# Gut microbiota-driven indole-3-propionic acid and kynurenine production is associated with improved metabolic adaptation in periparturient dairy cows

**DOI:** 10.1186/s40104-026-01497-6

**Published:** 2026-09-11

**Authors:** Xiaojing Liu, Zhantao Yang, Xinyue Zhang, Weixuan Tang, Yajing Wang, Zhijun Cao, Hongjian Yang, Wei Wang, Yangyi Hao, Shengli Li

**Affiliations:** 1 State Key Laboratory of Animal Nutrition and Feeding, Beijing Engineering Technology Research Center of Raw Milk Quality and Safety Control, College of Animal Science and Technology, China Agricultural University, Beijing, 100193 China; 2 Key Laboratory of Qinghai-Tibetan Plateau Animal Genetic Resource Reservation and Utilization, Ministry of Education, Southwest Minzu University, Chengdu, China

**Keywords:** Gastrointestinal microbiome, Metabolic regulation, Metagenome-assembled genomes, Microbial biotransformation, Tryptophan

## Abstract

**Background:**

Gastrointestinal microbes convert tryptophan into various bioactive metabolites that influence host energy metabolism; however, these mechanisms are not well understood in periparturient dairy cows, which experience marked metabolic challenges during this period.

**Results:**

In this study, we used periparturient dairy cows with rumen and ileal cannulas as in vivo models. Blood, rumen fluid, ileal digesta, and fecal samples were collected at four time points during the periparturient period. By combining metagenome-assembled genomes (MAGs) and targeted metabolite quantification, we characterized microbial tryptophan metabolism and associated metabolite profiles during the periparturient period. The results showed that postpartum cows exhibited significantly increased serum concentrations of triglyceride (TG), aspartate aminotransferase (AST), β-hydroxybutyrate (BHBA), and total bilirubin (T-Bil) compared with prepartum cows, together with decreased levels of several tryptophan metabolites, including indole-3-propionic acid (IPA) and kynurenine (KYN), indicating that tryptophan deficiency might aggravate metabolic disturbances. Metagenomic analysis identified 578 high-quality MAGs, of which 461 contained genes involved in microbial tryptophan metabolic pathways. Among these, the ruminal taxon *CAG-791* harbors *acdA* and contributes to IPA production, whereas the hindgut taxon *Treponema_D* harbors *kynB* and promotes KYN formation. Decreases in both taxa were consistent with the reduced levels of these metabolites observed above. In a follow-up in vivo trial with tryptophan supplementation, the abundance of *CAG-791* and *Treponema_D* increased, along with tryptophan-derived metabolites (IPA and KYN), which further partially mitigated metabolic disturbances.

**Conclusions:**

These findings characterize spatial and temporal changes in tryptophan metabolites and gut microbial features in periparturient dairy cows, and provide integrated evidence that alterations in tryptophan metabolism are associated with postpartum metabolic adaptation, thereby supporting the potential of tryptophan-targeted nutritional strategies to improve metabolic health in dairy cows.

**Supplementary Information:**

The online version contains supplementary material available at https://doi.org/10.1186/s40104-026-01497-6.

## Introduction

Microbiota-derived metabolites, including short-chain fatty acids, bile acids, and tryptophan, serve as key mediators of crosstalk between the gut microbiota and host metabolism [1]. Notably, tryptophan is an essential amino acid for protein synthesis and is metabolized via kynurenine, serotonin, and indole pathways into diverse bioactive metabolites that modulate host metabolism and influence host health [2, 3]. Among these metabolites, kynurenic acid helps maintain the energy balance in the adipose tissue by promoting the expression of genes involved in lipid metabolism, thermogenesis, and anti-inflammatory responses [4]. Moreover, indole-3-propionic acid (IPA) is associated with a low risk of type 2 diabetes, and supplementation alleviates glucose and lipid metabolism disorders [5]. In addition, in rats transitioning from pregnancy to lactation, high serotonin concentrations increase hepatic pyruvate carboxylase mRNA levels, which is a key control point for the entry of glucogenic precursors into gluconeogenesis [6]. Collectively, these findings indicate that all three tryptophan metabolic pathways produce metabolites that regulate host energy metabolism.

The periparturient window (approximately three weeks before to three weeks after calving) presents a considerable metabolic challenge for dairy cows. After calving, dry matter intake typically decreases, whereas the energy demand for lactation increases sharply, resulting in an energy deficit and metabolic disturbances [7]. Metabolic disturbances in dairy cows are associated with decreased conception rates, increased early embryonic mortality, and increased estrus silencing [8], reducing animal welfare and potentially causing stock and economic losses, all of which negatively affect farm profitability [9, 10]. Therefore, the periparturient period is a crucial window for precise nutritional and metabolic management in dairy cows.

Evidence indicates that gut microbiota modulates energy metabolism [11–15]. Tryptophan is an essential amino acid that cows obtain solely from their diet [16]. In the rumen, tryptophan participates in microbial protein synthesis and is efficiently metabolized by the rumen microbiome into multiple bioactive metabolites [17, 18]. Evidence from periparturient cows indicates that tryptophan metabolism is a key metabolic pathway associated with ketosis occurrence and development, and cows with ketosis display markedly reduced concentrations of tryptophan, kynurenine (KYN), and indole-3-acetic acid [19]. In addition, serotonin, a tryptophan-derived metabolite, regulates metabolic homeostasis during the periparturient period in dairy cows [20]. Accordingly, 5-hydroxytryptophan supplementation warrants further evaluation as a preventive strategy against subclinical and clinical hypocalcemia in dairy cows [21]. Collectively, these findings underscore the importance of tryptophan in periparturient energy metabolism.

The ruminant gastrointestinal tract is a continuous system of fermentation and nutrient absorption. Some dietary components are not fully degraded in the rumen and can enter the ileum and other distal intestinal segments for further digestion, generating a range of important metabolites that may influence nutrient utilization and host phenotype [22]. In addition, metagenomic evidence has shown that the functional pathways of cow hindgut microbiota include modules related to “tryptophan metabolism” that can be reshaped by dietary conditions, suggesting that tryptophan conversion is not limited to the rumen [23]. Moreover, microbial shifts have been associated with tryptophan-derived metabolites (e.g., indole-3-acetic acid), further supporting the potential role of the hindgut in generating these metabolites and mediating their effects on the host [24]. Nevertheless, previous studies have largely focused on the rumen, and our understanding of ruminal microbiota and their metabolic features is relatively advanced. In contrast, limited studies that have explored other intestinal segments and have usually relied on samples collected at slaughter, providing only single-time-point, cross-sectional snapshots and lacking systematic analysis of how the gut changes over time. Recent evidence suggests that ruminal microbial communities and their functional profiles exhibit temporal oscillations, indicating that microbiome measurements are not static [25]. The periparturient period in dairy cows is crucial when the gut microbiota and host physiology undergo substantial and coordinated remodeling. Moreover, systematic data on intestinal tryptophan metabolism in cows, periparturient shifts in its metabolites, the microbial contributors involved, and how these processes couple with host energy metabolism are needed. Thus, longitudinal investigations of tryptophan metabolism and microbial responses across multiple intestinal segments are essential to comprehensively understand the regulatory role of tryptophan in the energy metabolism of periparturient dairy cows.

To address these gaps, this study aimed to clarify the role of tryptophan metabolism in the metabolic adaptation of periparturient cows by using dairy cows fitted with rumen and ileal cannulas for a comprehensive evaluation. We proposed three questions: (1) whether tryptophan-derived metabolites showed temporal shifts in the gastrointestinal tract of transition cows from prepartum to postpartum, and whether different gut regions exhibited similar or distinct patterns of change in these metabolites? (2) what the key genes and taxa involved in tryptophan metabolism were, and how they were associated with metabolic disturbances in periparturient cows? (3) whether exogenous tryptophan supplementation could remodel the metabolite profile and improve metabolic adaptation, and if so, which functional mediators and microbial taxa were involved? We hypothesized that inadequate tryptophan supply postpartum would reduce tryptophan and its metabolites, thereby contributing to metabolic disturbances, whereas supplementation would shift key metabolites and mitigate these alterations. We integrated time-resolved targeted tryptophan metabolomics of rumen, ileum, feces, and plasma, along with genome-resolved metagenomics of rumen, ileum, and feces to map the spatiotemporal distribution of metabolites and pathway-encoding microbes. Additionally, a 21-d postpartum tryptophan supplementation experiment was conducted to evaluate its functional effects on metabolic adaptation. Overall, our findings may enable more precise nutritional interventions, thereby alleviating metabolic disturbances and ultimately supporting dairy cow productivity and animal welfare.

## Materials and methods

### Animal ethics statement

All animal procedures were approved by the Institutional Animal Care and Use Committee of China Agricultural University (protocol numbers: AW42504202-1-2, AW40115202-1-01).

### Animals, feeding, experimental design, and sample collection

This study was conducted at Yuexiu Huishan Dairy Research Farm in Shenyang, China. The experimental animals were five multiparous Holstein dairy cows fitted with ruminal and ileal cannulas and maintained in our research program (parity, 2.5 ± 0.5; body weight, 680 ± 36 kg). Detailed information on cannula types, implantation sites, and surgical and post-operative care procedures has been reported in our previous study [26]. All cows had fully recovered and were clinically healthy before the start of the experiment. Each cow was housed separately and fed the experimental diets ad libitum twice daily, with the morning feeding provided immediately after the morning milking and the second feeding provided at 15:00 h. Water was available ad libitum via individual water troughs. All cows were milked twice daily at 08:00 and 20:00 h using a hand-pushed milking machine (Zibo Zhisong Electric Motor Co., Ltd., China). The feed ingredients and nutrient compositions are presented in Table S1, and the formula was prepared according to the nutrient requirements of dairy cattle [16].

On d 7 prepartum and d 7, 14, and 21 postpartum (D−7, D7, D14, and D21), cows were milked first and then fed in the morning. At each time point, all samples (blood, rumen fluid, ileal digesta, and feces) were collected sequentially during a single sampling session approximately 2 h after the morning feeding. To standardize the sampling procedure, blood samples were collected from all cows first, followed by rumen fluid, ileal digesta, and fecal samples. The same sampling order was applied at each sampling time point. Blood samples (10 mL) were collected from the coccygeal vein. Blood samples in no-anticoagulant tubes were allowed to clot at ambient temperature (20–25 °C) for 30 min and then centrifuged at 3,000 × *g* for 10 min to obtain serum, which was stored at −20 °C for biochemical analysis. EDTA-anticoagulated blood was immediately centrifuged at 3,000 × *g* for 10 min to obtain plasma, which was stored at −80 °C for targeted tryptophan metabolite analysis. Rumen fluid and ileal digesta were collected through the rumen and ileal cannulas, respectively. For each rumen fluid and ileal digesta sample, two separate aliquots were transferred into individual 2-mL cryogenic tubes and immediately stored in liquid nitrogen. One aliquot was used for targeted tryptophan metabolite analysis, and the other was used for microbial metagenomic sequencing. Fecal samples were collected directly from the rectum by research personnel wearing sterile long-arm gloves. After thorough mixing, two separate portions of each fecal sample were transferred into individual 2-mL cryogenic tubes and immediately stored in liquid nitrogen. One portion was used for targeted tryptophan metabolite analysis, and the other was used for microbial metagenomic sequencing.

A tryptophan supplementation experiment was conducted on another commercial dairy farm (37°29′N, 114°54′E, Xingtai, China), which housed approximately 8,000 Holstein dairy cows. A total of twelve cows with similar backgrounds (parity, 2–3; body weight, 700 ± 38 kg) were selected and randomly assigned to two groups. The cows in the control group were fed a total mixed ration (TMR) without tryptophan supplementation (CON), while the cows in the treatment group were fed a TMR with tryptophan supplementation (100 g/d) via oral drench from d 0 to d 21 after calving (TRP). Additionally, cows in the CON group were drenched with normal saline to maintain the same management conditions as the TRP group. The feed ingredients and nutrient compositions are listed in Table S2.

On d 21 after calving, blood, rumen fluid, and fecal samples were collected approximately 2 h after the morning feeding. Blood samples were collected from the coccygeal vein, as described for the first animal experiment, followed by rumen fluid collection via oral intubation and fecal sample collection from the rectum. Rumen fluid samples were collected via oral intubation (Wuhan Anscitech Animal Husbandry Technology Co., Ltd., Wuhan, China) using a 50-mL syringe (Beijing Hua Xia Heng Yuan Technology Co., Ltd.). The initial 150 mL of rumen content was discarded to avoid saliva contamination, and two aliquots were stored in 2-mL cryogenic tubes in liquid nitrogen for targeted analysis of tryptophan metabolites and full-length 16S rRNA gene sequencing. Fecal samples were obtained from the rectum by research personnel wearing sterile long-arm gloves, immediately transferred to 2-mL cryogenic tubes, and stored in liquid nitrogen until subsequent analysis. Measurements of serum indices related to energy metabolism and liver function, as well as quantification of tryptophan metabolites in the plasma, rumen content, and feces, were performed using the same post-collection analytical procedures as those used in the first animal experiment. Milk yield was recorded daily from calving to 21 d postpartum. The milk production at each milking was automatically recorded using the ALPRO™ system (DeLaval). On 21 d postpartum, milk samples were collected at 07:00, 14:00, and 20:00 h through the ALPRO™ milk sampling device and pooled at a ratio of 4:3:3. A 50-mL aliquot of the composite milk sample was preserved with bronopol and stored at 4 °C until analysis. The milk samples were sent to the Beijing Dairy Cow Center for milk composition analysis, including milk fat, milk protein, and lactose. Milk composition was determined using a near-infrared reflectance spectroscopy analyzer (Seris300 CombiFOSS; Foss Electric), which integrates the MilkoScan (Foss Electric) module for milk composition analysis and the Fossomatic (Foss Electric) module for flow cytometric somatic cell counting.

### Measurement of serum biochemical indicators

The concentrations of alanine aminotransferase (ALT), high-density lipoprotein (HDL), blood urea nitrogen (BUN), non-esterified fatty acids (NEFA), total cholesterol (TC), triglyceride (TG), aspartate aminotransferase (AST), glucose (GLU), low-density lipoprotein (LDL), β-hydroxybutyrate (BHBA), total bilirubin (T-Bil), and direct bilirubin (D-Bil) were determined using a fully automatic biochemical analyzer (GF-D200, Gaomi Analytical Instrument Co., Ltd., Gaomi, China) and commercial kits (Nanjing Jiancheng Bioengineering Institute, Nanjing, China).

### Targeted metabolomics

For targeted quantification of tryptophan metabolites in rumen fluid, ileal digesta, fecal samples, and plasma, samples were thawed on ice to minimize metabolite degradation. For rumen fluid, ileal digesta, and plasma samples, 50 μL of each sample was transferred into a 1.5-mL centrifuge tube and mixed with 300 μL of acetonitrile containing internal standards. The mixture was shaken at 1,000 r/min for 10 min at 4 °C and then extracted at −20 °C for 30 min. For fecal samples, 10 mg of lyophilized fecal sample was weighed into a 1.5-mL centrifuge tube, mixed with zirconia beads and 45 μL of water, and homogenized for 3 min using a BB24 homogenizer (Next Advance, Inc., NY, USA). Then, 300 μL of acetonitrile containing internal standards was added, and the mixture was homogenized again for 3 min, followed by extraction at −20 °C for 30 min. After extraction, all samples were centrifuged at 18,000 × *g* for 20 min at 4 °C (Microfuge 20R, Beckman Coulter, Inc., IN, USA). A 300-μL aliquot of the supernatant was transferred to a new centrifuge tube and dried under vacuum at 4 °C. The residue was reconstituted with 100 μL of 0.1% formic acid in water, shaken at 1,000 r/min for 10 min at 4 °C, and centrifuged at 18,000 × *g* for 10 min at 4 °C. Finally, 90 μL of the supernatant was transferred to a sealed 96-well plate for LC–MS analysis. Mixed tryptophan standards, including KYN, indole-3-lactic acid, and indole-3-acetic acid, were prepared in acetonitrile and processed in parallel. Quantitative analysis of tryptophan metabolites was performed using a Metabo-Profile targeted platform (ACQUITY UPLC-Xevo TQMS; Waters Corp., Milford, MA, USA) [27]. Chromatographic separation was performed using an ACQUITY UPLC HSS T3 column (1.8 μm, 2.1 mm × 100 mm) with an HSS T3 VanGuard guard column (1.8 μm, 2.1 mm × 5 mm). The mobile phases were 0.1% (v/v) formic acid in water and 0.1% (v/v) formic acid in acetonitrile. Each batch of samples was analyzed in parallel with a reagent blank and a quality control (QC) sample. The QC sample was prepared by pooling aliquots of all samples and was injected once after every 10 sample injections. Raw data were processed using MassLynx software (version 4.1; Waters, Milford, MA, USA) for component extraction, peak integration, calibration, and quantification.

### Metagenome assembly and binning

#### DNA extraction, library preparation, sequencing, and read processing

Total genomic DNA from rumen fluid, ileal digesta, and fecal samples was extracted using the Mag-Bind^®^ Soil DNA Kit (Omega Bio-tek, Norcross, GA, USA) according to the manufacturer’s protocol. DNA concentration and purity were measured using a Nanodrop 2000 spectrophotometer, and DNA quality and integrity were verified by electrophoresis on 1% agarose gels. Genomic DNA from each individual sample was used separately for sequencing library construction. Genomic DNA was fragmented using Covaris M220 (Gene Company Limited, China), and ~ 350-bp fragments were size-selected for paired-end (PE) library preparation using the NEXTFLEX Rapid DNA-Seq kit (Bioo Scientific, USA). During library preparation, each library was ligated with a unique sample-specific index sequence to enable sample identification and demultiplexing after sequencing. After library quality assessment, equimolar amounts of the indexed libraries were pooled and sequenced together on a single high-throughput flow cell using the Illumina NovaSeq X Plus platform (Illumina, USA).

Raw reads were processed using fastp v0.23.1 [28] (https://github.com/OpenGene/fastp) to remove adapter sequences and discard reads with a post-trim length of < 50 bp or mean base quality of < Q20, retaining high-quality reads. Clean reads were aligned to the bovine genome using BWA [29] (http://bio-bwa.sourceforge.net, v0.7.9a) to remove host gene contamination. Reads assigned to metazoans or Viridiplantae were also removed for downstream analysis. In total, 9,060,679,618 raw reads were generated from all metagenomic samples, with a mean sequencing depth of 151,011,327 ± 1,655,127 reads per sample (mean ± SEM). After adapter removal and quality filtering, 8,995,130,796 quality-filtered reads were retained, corresponding to 149,918,847 ± 1,645,337 reads per sample. The quality-filtered reads were subsequently mapped against the bovine reference genome to remove host-derived sequences. Ultimately, 8,658,576,630 host-depleted clean reads were obtained, corresponding to 144,309,611 ± 2,405,979 reads per sample, and were used for metagenomic assembly. Detailed sequencing statistics for each sample are provided in Table S3.

#### Metagenomic assembly, genome binning, and quality assessment

For each sample, clean reads were assembled de novo using MEGAHIT v1.1.2 [30] (https://github.com/voutcn/megahit) and contigs shorter than 500 bp were discarded. Contigs ≥ 1,000 bp from each assembly were subjected to single-sample binning using MetaBAT2 v2.12.1 [31] (https://bitbucket.org/berkeleylab/metabat), CONCOCT v0.5.0 [32] (https://github.com/BinPro/CONCOCT), and MaxBin2 v2.2.5 [33] (https://sourceforge.net/projects/maxbin/). The binning results were integrated with the DAS Tool v1.1.0 [34] (https://github.com/cmks/DAS_Tool) to obtain a consensus set of bins, which were further curated with RefineM v0.0.24 [35] (https://github.com/wwood/RefineM). All curated bins from all samples were pooled, and genome quality was assessed using CheckM [36] (https://github.com/Ecogenomics/CheckM/wiki) to estimate completeness, contamination, and strain heterogeneity. Bins with completeness ≥ 90% and contamination < 5% were retained. High-quality bins were dereplicated using dRep v3.4.2 [37] at an estimated average nucleotide identity (ANI) of ≥ 99% (strain level), retaining the best-quality representative genome per cluster. The resulting nonredundant catalog constituted the final metagenome-assembled genomes (MAGs). Taxonomic classification of MAGs was performed using GTDB-Tk v2.3.0 [38]. Quality-controlled reads were mapped to the MAG catalog using CoverM v0.6.1 to quantify per-sample abundance (TPM). Each MAG then underwent de novo gene prediction to annotate the coding sequences, tRNAs, and rRNAs. Protein-coding sequences were predicted using Prodigal v2.6.3 [39] (https://github.com/hyattpd/Prodigal), yielding nucleotide and amino acid sequences for downstream functional and phylogenetic analyses. Tandem repeats were identified using Tandem Repeats Finder v4.07b [40] (http://tandem.bu.edu/trf/trf.html). Functional annotation was performed by aligning MAG-derived proteins to the NCBI nonredundant database (NR; nr_202410) using DIAMOND v0.8.35 [41] (https://github.com/bbuchfink/diamond) with an e-value cutoff of 1 × 10^−5^, transferring annotations from the best hits. The same protein set was aligned to eggNOG and KEGG with Diamond v0.8.35 (e-value ≤ 1 × 10^−5^) to assign Clusters of Orthologous Genes (COG) categories, Kyoto Encyclopedia of Genes and Genomes (KEGG) orthologs, and pathways. These annotations were used for downstream functional classification and pathway enrichment analyses [42]. Phylogenetic trees were generated using PhyloPhlAn [43] (v.1.0) and visualized using iTOL [44] (v.4.3.1).

#### Spike-in-based absolute quantification of ruminal and fecal microbiota

Absolute quantification of rumen and feces microbiota of tryptophan supplementation experiment based on the 16S rRNA gene was performed as follows: Total DNA was extracted from rumen and fecal samples using the E.Z.N.A. Soil DNA Kit (Omega Bio-tek, Norcross, GA, USA) according to the manufacturer’s instructions. A synthetic full-length 16S rRNA gene spike-in standard (conserved 16S rRNA regions with synthetic variable regions) was added to each DNA extract at a pre-set amount as an internal reference for absolute quantification. Spike-in-amended DNA was used as a template to amplify the full-length bacterial 16S rRNA gene using the 8-bp barcoded universal primers 27 F (5′-AGAGTTTGATCMTGGCTCAG-3′) and 1492R (5′-CGGTTACCTTGTTACGACTT-3′), and a positive control was used [45]. Amplicons were used to prepare barcoded SMRTbell libraries following the manufacturer’s instructions. Libraries from 24 uniquely barcoded samples were pooled in equimolar amounts and sequenced on a PacBio Revio platform in the HiFi mode. Circular consensus sequencing (CCS/HiFi) reads were generated and demultiplexed by sample-specific barcodes using SMRT Link, and the reads were quality-filtered by length and quality scores. Quality-filtered CCS reads were processed in QIIME 2, where denoising, error correction, and chimera removal were performed using DADA2 to infer the amplicon sequence variants (ASVs). For taxonomic assignment, ASVs were classified in QIIME 2 using a naïve Bayes classifier trained on a GTDB-derived 16S rRNA reference dataset, which was chosen to maintain taxonomic consistency with the MAG classification based on GTDB-Tk. Because spike-ins are not part of the native microbial community, spike-in ASVs were identified in the ASV table, quantified, and removed from downstream community composition analyses. The known spike-in input copy number and its observed read counts were used to derive sample-specific scaling factors, which were applied to convert the ASV relative abundances into absolute copy numbers for each ASV per sample and to estimate the total microbial load per sample for subsequent absolute quantification analyses.

### Statistical analysis

Data are expressed as the means ± standard error of the mean (SEM). Statistical analyses were performed using SPSS (version 22.0; IBM, Armonk, NY, USA) and GraphPad Prism 9.0 (GraphPad, San Diego, CA, USA). In the rumen- and ileal-cannulated cow experiment, with four repeated time points per cow, the analyses were performed separately for each segment (rumen, ileum, and feces). Normally distributed variables were analyzed using repeated-measures ANOVA with Bonferroni-adjusted pairwise comparisons. Microbiome and gene variables were analyzed using the Friedman test; when appropriate, post-hoc pairwise Wilcoxon signed-rank tests with Bonferroni adjustment were performed. For multi-feature testing (e.g., taxa or genes), *P* values were further corrected using the Benjamini–Hochberg false discovery rate (FDR) within each segment. For the validation experiment comparing two independent groups, normally distributed data were analyzed using the independent-samples *t*-test and non-normally distributed data were analyzed using the Mann–Whitney U-test. A two-sided α of 0.05 was used. Spearman’s correlation analysis was conducted and visualized using the CNS knowall cloud platform (https://cnsknowall.com). Random Forest analysis was performed to identify the important indicators of tryptophan metabolites using the R rfPermute package (v.2.5.1) [46]. *P* < 0.05 was considered significantly different.

## Results

### Temporal dynamics of systemic metabolism in peripartal dairy cows

**Fig. 1:**
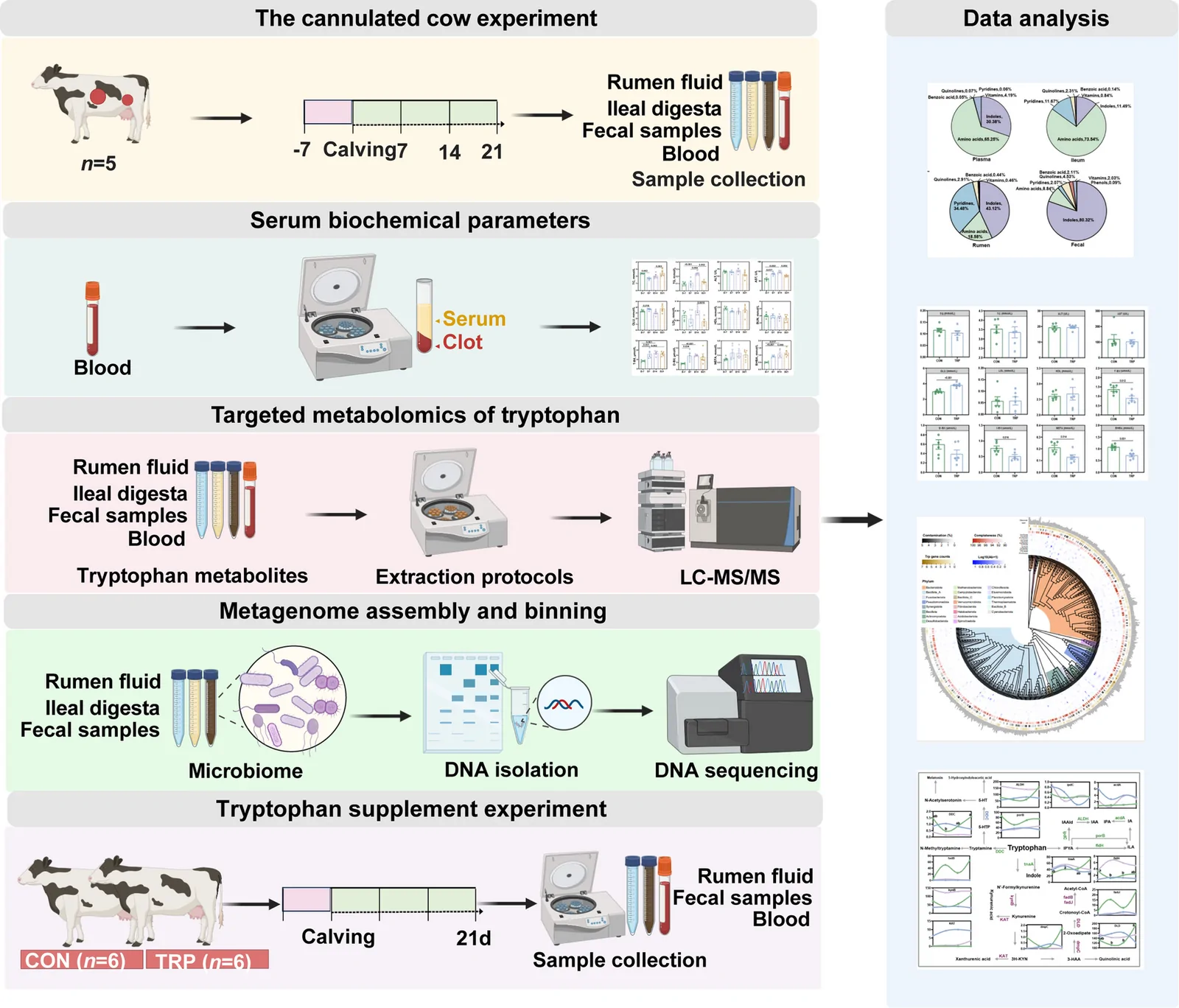
Overview of the experimental design, sample collection, and multi-omics analytical workflow. In the longitudinal cannulated cow experiment, five dairy cows fitted with rumen and ileal cannulas were sampled at d 7 before calving and d 7, 14, and 21 after calving (D−7, D7, D14, and D21). Rumen fluid, ileal digesta, fecal samples, and blood samples were collected. Rumen fluid, ileal digesta, feces, and plasma were used for targeted tryptophan metabolomics. Rumen fluid, ileal digesta, and fecal samples were used for microbial metagenomic sequencing, assembly, and genome binning. In a separate tryptophan supplementation experiment, twelve postpartum dairy cows were assigned to a control group (CON, *n* = 6) or a tryptophan-supplemented group (TRP, *n* = 6). Rumen fluid, fecal samples, and blood samples were collected on postpartum d 21. Blood samples were used for serum biochemical analysis, whereas rumen fluid and fecal samples were used for targeted tryptophan metabolite analysis and full-length 16S rRNA gene sequencing. The right panel summarizes the major downstream data analyses, including metabolite composition analysis and integrated visualization of microorganisms encoding genes involved in tryptophan metabolic pathways

**Fig. 2:**
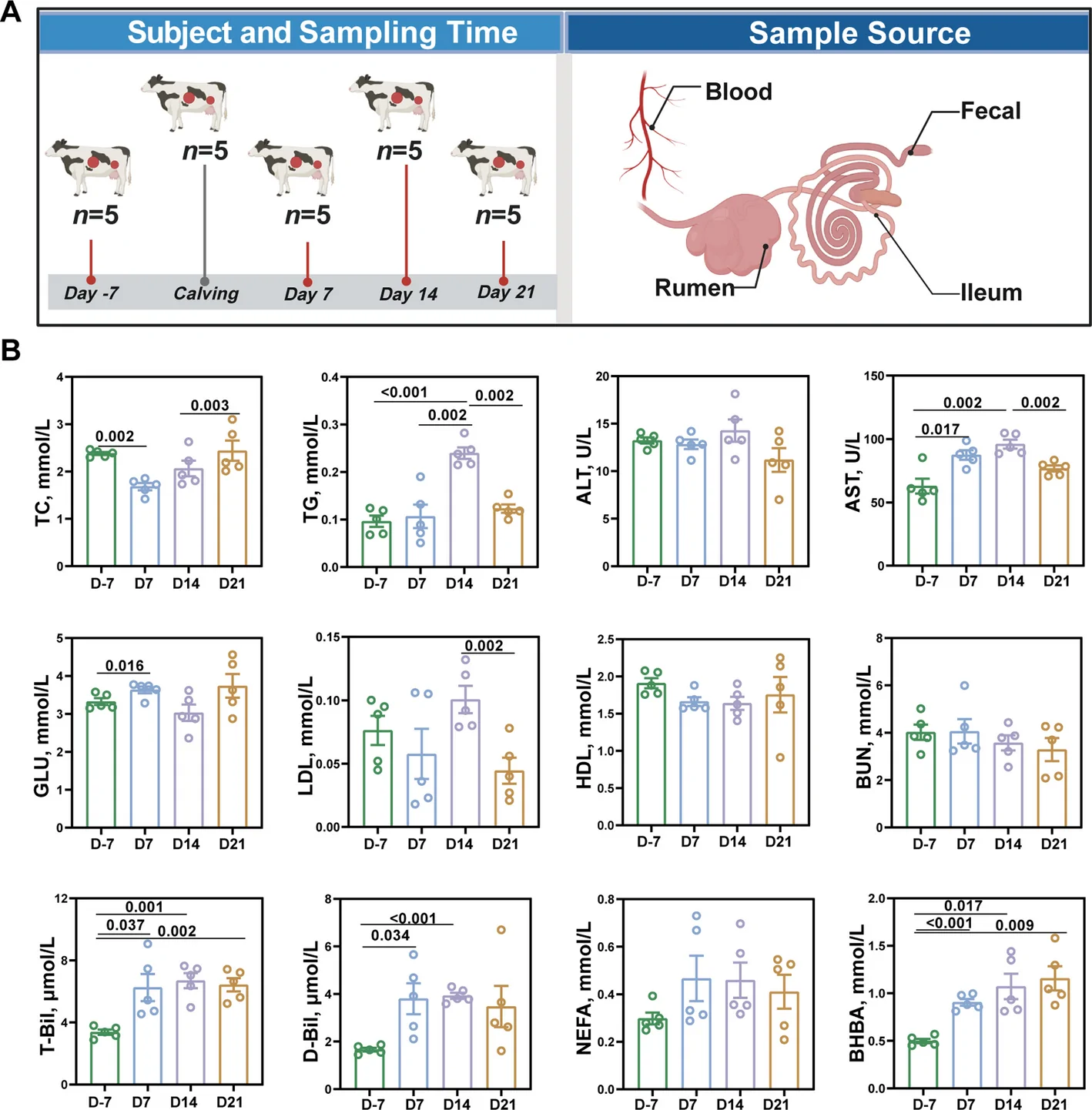
Changes in serum energy‐metabolism and liver function indices during the periparturient period. **A** Experimental animal, sampling time points, and sample sources for the first animal experiment. **B** Longitudinal changes in serum energy‐metabolism and liver function indices during the periparturient period, including β-hydroxybutyrate (BHBA), glucose (GLU), non-esterified fatty acids (NEFA), triglyceride (TG), total cholesterol (TC), aspartate aminotransferase (AST), alanine aminotransferase (ALT), low-density lipoprotein cholesterol (LDL), high-density lipoprotein cholesterol (HDL), blood urea nitrogen (BUN), total bilirubin (T-Bil), and direct bilirubin (D-Bil). Data are presented as mean ± standard error of the mean (SEM). D−7, D7, D14, and D21 represent 7 d prepartum and 7, 14, and 21 d postpartum, respectively; the same abbreviations are used throughout

This study consisted of longitudinal sample collection, serum biochemical analysis, targeted profiling of tryptophan metabolites, metagenomic analysis at the genome level, and a subsequent tryptophan supplementation experiment (Fig. 1). In the cannulated cow experiment, blood, rumen, ileal, and fecal samples were collected at 7 d before calving (D−7) and at 7, 14, and 21 d after calving (D7, D14, and D21, respectively) (Fig. 2A). To examine the periparturient dynamics in energy metabolism, serum biochemical indices were compared at D−7, D7, D14, and D21. The result showed that the concentrations of ALT, HDL, BUN, and NEFA did not change significantly over time (*P* > 0.05). In contrast, the concentrations of TC, TG, AST, GLU, LDL, BHBA, T-Bil, and D-Bil showed significant temporal variations. The TC concentration was significantly lower at D7 than at D−7, and significantly higher at D21 than at D14 (*P* < 0.05). The TG concentrations at D14 were significantly higher than those at D−7, D7, and D21 (*P* < 0.05). Similarly, the AST concentrations were higher at D7 and D14 than at D−7, and were also higher at D14 than at D21 (*P* < 0.05). The GLU concentration at D7 was significantly higher than at D−7 (*P* < 0.05). The LDL concentration at D14 was higher than at D21 (*P* < 0.05). Additionally, the concentrations of postpartum T-Bil and BHBA were higher than those in the prepartum period, and the concentrations of D-Bil at D7 and D14 were higher than those in the prepartum period (*P* < 0.05; Fig. 2B). Collectively, these results indicate that periparturient metabolic dynamics are characterized by increased lipid mobilization, reflecting the metabolic disturbances that occur during the postpartum period.

### Temporal dynamics in systemic and gastrointestinal tryptophan metabolic profiles in peripartal dairy cows

**Fig. 3:**
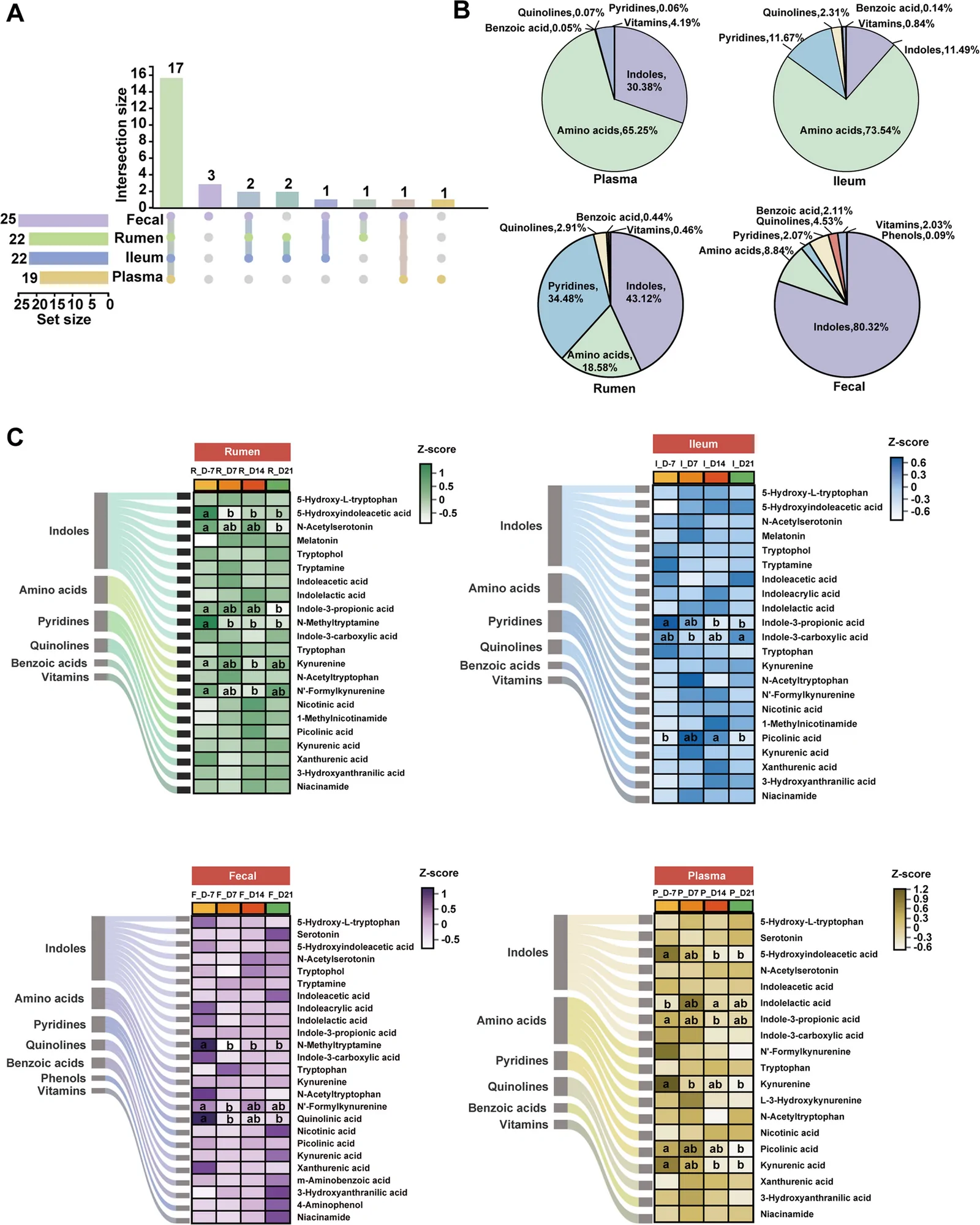
Distribution of tryptophan metabolites in the gastrointestinal and blood of periparturient dairy cows. **A** The UPSET diagram shows the number of distinct tryptophan metabolites shared across different sites. **B** Proportions of tryptophan metabolites in the plasma, ileum, rumen, and feces contents of periparturient cows. **C** Temporal dynamics of tryptophan metabolites across compartments. The Sankey diagram depicts metabolite class assignments. For each compartment, including rumen fluid (R_D−7, R_D7, R_D14, and R_D21), ileal digesta (I_D−7, I_D7, I_D14, and I_D21), feces (F_D−7, F_D7, F_D14, and F_D21), and plasma (P_D−7, P_D7, P_D14, and P_D21), heat maps show row-wise Z-scores across four periparturient time points. R, I, F, and P represent rumen fluid, ileal digesta, feces, and plasma, respectively, whereas D−7, D7, D14, and D21 represent 7 d before calving and 7, 14, and 21 d after calving, respectively. Different letters denote significant differences among time points for the same metabolite (*P* < 0.05)

Tryptophan metabolism is linked to metabolic dysregulation. Owing to the significant metabolic differences in periparturient dairy cows, we performed targeted metabolomics of the tryptophan pathway in rumen fluid (R_D−7, R_D7, R_D14, R_D21), ileal digesta (I_D−7, I_D7, I_D14, I_D21), feces (F_D−7, F_D7, F_D14, F_D21), and plasma (P_D−7, P_D7, P_D14, P_D21). We measured 19, 22, 22, and 25 tryptophan-derived metabolites in the plasma, ileum, rumen, and feces, respectively. Overall, these metabolites represent three tryptophan pathways (kynurenine, serotonin, and indole). Of these, 17 metabolites were found in all four biological matrices. Three metabolites were unique to feces (3-aminobenzoic acid, 4-aminophenol, quinolinic acid) and one to plasma (L-3-hydroxykynurenine). No unique metabolites were detected in the rumen or the ileum (Fig. 3A). Among the detected metabolites, amino acid-related compounds made up over 60% in the plasma and ileum, followed by indole derivatives; in the rumen, indoles (43.12%), pyridine derivatives (34.48%), and amino acids (18.58%) were the most common; and in feces, indoles accounted for over 80%, followed by amino acid–related compounds (8.84%) (Fig. 3B).

To assess the temporal variation in tryptophan metabolites across regions, repeated-measures ANOVA with Bonferroni-adjusted pairwise comparisons was performed. In the rumen, compared with R_D−7, the concentrations of 5-hydroxyindoleacetic acid and N-methyltryptamine were significantly lower at R_D7, R_D14, and R_D21, those of N-acetylserotonin and IPA were significantly lower at R_D21, and those of KYN and N′-formylkynurenine were significantly lower at R_D14 (*P* < 0.05). In feces, the concentrations of N-methyltryptamine were significantly lower postpartum than prepartum. The concentration of N′-formylkynurenine decreased at F_D7 relative to F_D−7, and the concentration of quinolinic acid was significantly reduced at both F_D7 and F_D21 compared with F_D−7 (*P* < 0.05). In the ileum, the concentration of IPA was significantly lower at I_D14 and I_D21 than at I_D−7. The concentration of indole-3-carboxylic acid increased at I_D21 and was higher than that at I_D7, whereas the concentration of picolinic acid peaked at I_D14, exceeding the levels at I_D21 and I_D−7 (*P* < 0.05). In the plasma, the concentrations of 5-hydroxyindoleacetic acid and kynurenic acid were significantly lower at P_D14 and P_D21 than at P_D−7. KYN and picolinic acid concentrations were also reduced at P_D21, and KYN concentrations were significantly decreased at P_D7 relative to P_D−7. The IPA concentrations were significantly lower at P_D14 than at P_D−7. In contrast, indole-3-lactic acid levels were significantly higher in P_D14 than in P_D−7 (Fig. 3C). Together, these results indicate that tryptophan-derived metabolite profiles differ across systemic and gastrointestinal compartments in periparturient dairy cows, and that a subset exhibits temporal dynamics. Metabolites were identified as differentially displaying specific regional patterns.

### Correlations between tryptophan metabolites and serum biochemical parameters

**Fig. 4:**
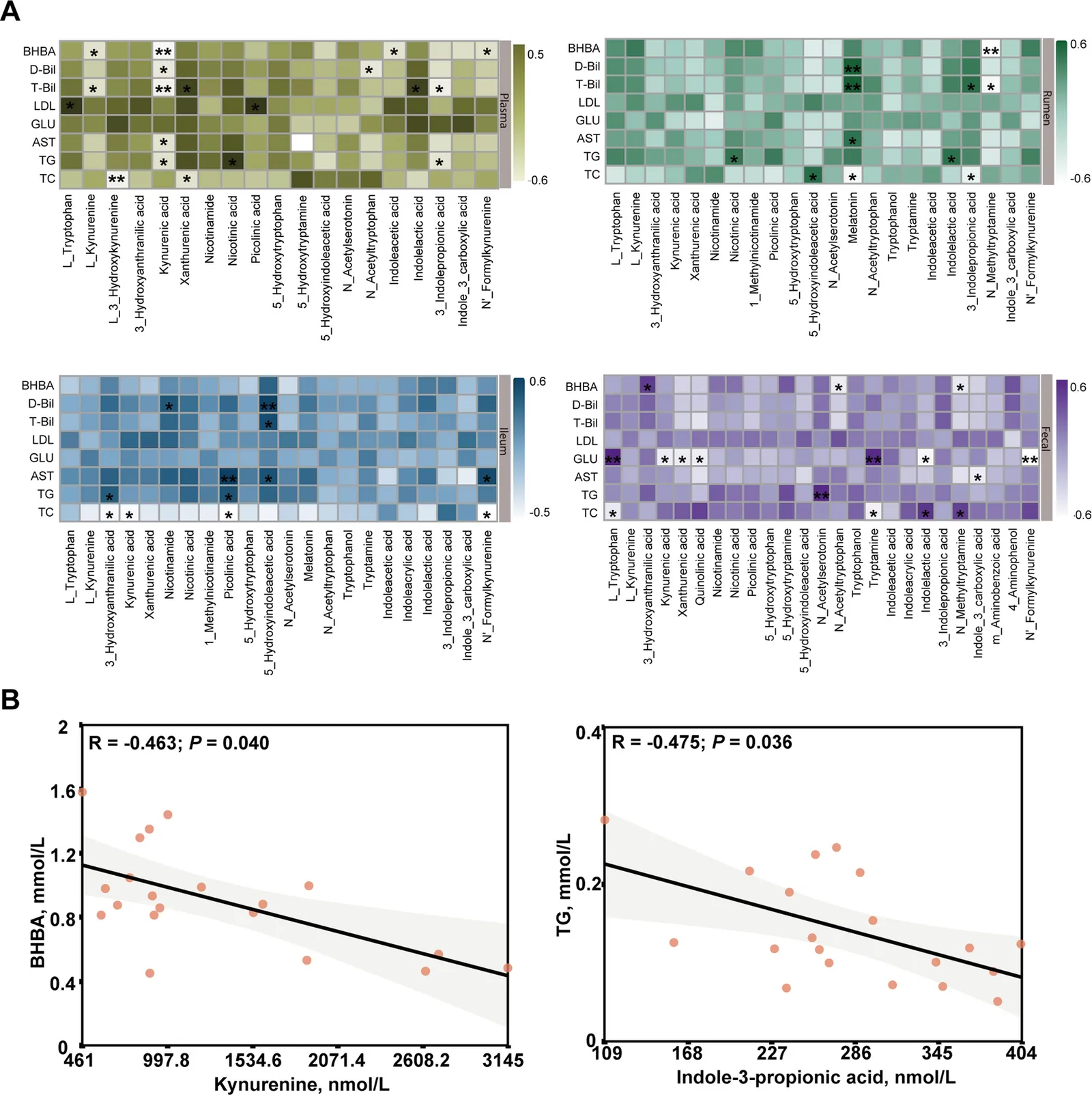
Associations between tryptophan metabolites and different serum indices of energy metabolism and liver function. **A** Compartment-specific heat maps (rumen, ileum, feces contents, plasma) showing correlations between metabolites and serum indices of energy metabolism and liver function; ^*^P < 0.05; ^**^P < 0.01. **B** Scatterplots illustrating two key associations: β-hydroxybutyrate (BHBA) with kynurenine (KYN), and triglyceride (TG) with indole-3-propionic acid (IPA)

To assess the link between tryptophan metabolites and host energy metabolism, we performed Spearman’s rank correlations in specific regions between tryptophan-derived metabolites and a subset of serum biochemical indicators that differed over time. Significant associations were observed for each biological matrix (*P* < 0.05). In the plasma, kynurenic acid was significantly negatively associated with BHBA, D-Bil, T-Bil, AST, and TG (*P* < 0.05). In the rumen, melatonin was significantly positively correlated with D-Bil, T-Bil, and AST but negatively correlated with TC (*P* < 0.05). In the ileum, 5-hydroxyindoleacetic acid was positively correlated with D-Bil, T-Bil, and AST, whereas picolinic acid was positively correlated with AST and TG, but negatively correlated with TC (*P* < 0.05). In feces, glucose was positively correlated with tryptophan and tryptamine, but negatively correlated with kynurenic acid, xanthurenic acid, quinolinic acid, N′-formylkynurenine, and indolelactic acid (*P* < 0.05) (Fig. 4A; Table S4). Given that serum TG and BHBA are representative indicators related to energy metabolism, we further focused on tryptophan metabolites associated with these two parameters. Notably, IPA and KYN showed significant negative correlations with serum TG and BHBA levels (Fig. 4B), highlighting their potential relevance to systemic energy status. The random forest analysis consistently ranked IPA and KYN among the most important metabolites (Fig. S1).

### Temporal dynamics of microbial gene abundance related to tryptophan metabolism across gastrointestinal in periparturient dairy cows

**Fig. 5:**
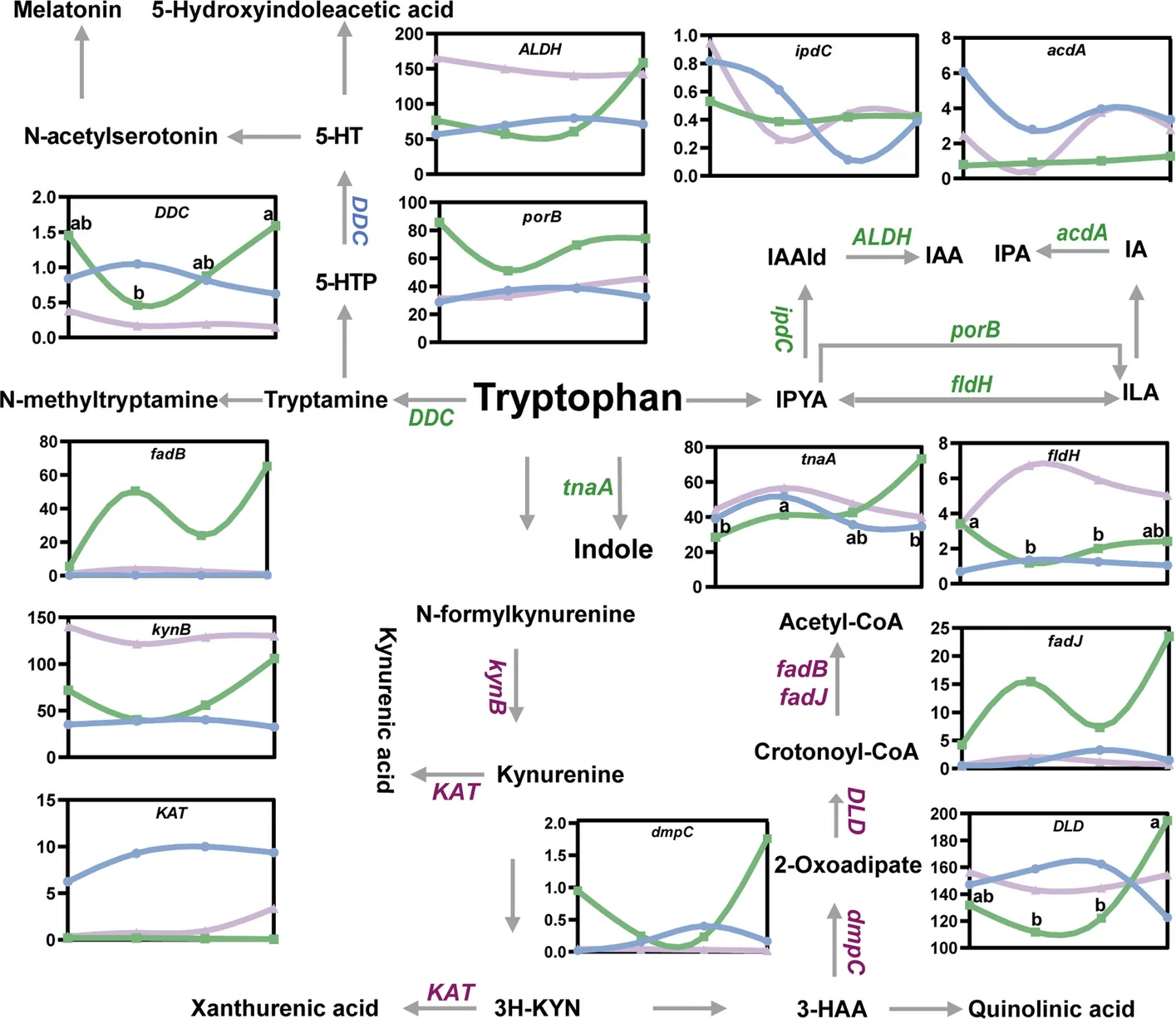
Changes in genes involved in microbial tryptophan metabolism across gastrointestinal regions. Different letters indicate significant differences in gene abundances across time points (D−7, D7, D14, and D21) (*P* < 0.05). Green denotes rumen, blue denotes ileum, and purple denotes feces. ILA, indole-3-lactic acid; IPA, indole-3-propionic acid; IAA, indole-3-acetic acid; IPYA, indole-3-pyruvic acid; 5-HT, 5-hydroxytryptamine; 5-HTP, 5-hydroxytryptophan; IAAld, indole-3-acetaldehyde; IA, indole-3-acrylic acid; 3-HAA, 3-hydroxyanthranilic acid; 3H-KYN, 3-hydroxykynurenine

Building on the finding that tryptophan metabolic pathways are microbially mediated and that numerous tryptophan metabolites are significantly correlated with serum metabolic indicators, we investigated microbial mechanisms by performing shotgun metagenomic sequencing on 60 gastrointestinal content samples collected from five cows in three regions (rumen, ileum, and feces) and four time points (d 7 prepartum; d 7, 14, and 21 postpartum) to quantify the abundance and temporal dynamics of tryptophan metabolism genes. First, we characterized metagenome-derived genes related to tryptophan metabolism across different biological matrices and time points in periparturient cows. A total of 13 tryptophan metabolism-related genes were detected in the metagenomic dataset, including *DDC*, *ALDH*, *ipdC*, *fldH*, *acdA*, *tnaA*, *porB*, *kynB*, *KAT*, *dmpC*, *DLD*, *fadB*, and *fadJ*. Among these genes, most were detected across all biological matrices and time points, whereas *KAT* was not detected in rumen and fecal samples at d 7 prepartum. We then assessed the overall abundance patterns and temporal dynamics of these tryptophan metabolism-related genes (Fig. S2A). The total abundance of these genes showed no significant temporal variation in the rumen or feces, whereas the ileal abundance at d 21 postpartum was significantly higher than that at d 7 and 14 postpartum (Fig. S2B). Across biological matrices, these genes were mainly assigned to the indole and kynurenine pathways, whereas relatively lower abundance was mapped to the serotonin pathway (Fig. S2C). Tryptophan metabolism genes were mapped to the pathway, and the temporal dynamics of their specific regions were profiled using line plots. The ruminal *tnaA* gene showed significant temporal changes (*P* < 0.05), peaking on postpartum d 7, with levels higher than those on prepartum d 7 and postpartum d 21. In the ileum, *DDC*, *fldH*, and *DLD* genes exhibited notable fluctuations (*P* < 0.05), reaching their lowest values on postpartum d 7; *DDC* and *DLD* genes on postpartum d 7 were lower than those on postpartum d 21, and *fldH* gene on postpartum d 7 was below that on prepartum d 7. No significant differences were observed in fecal gene abundance (*P* > 0.05; Fig. 5). Overall, these findings indicate the prevalence and specificity of tryptophan metabolism in the gastrointestinal microbiome of periparturient dairy cows.

### MAGs are involved in tryptophan metabolism pathways in the gastrointestinal of periparturient dairy cows

**Fig. 6:**
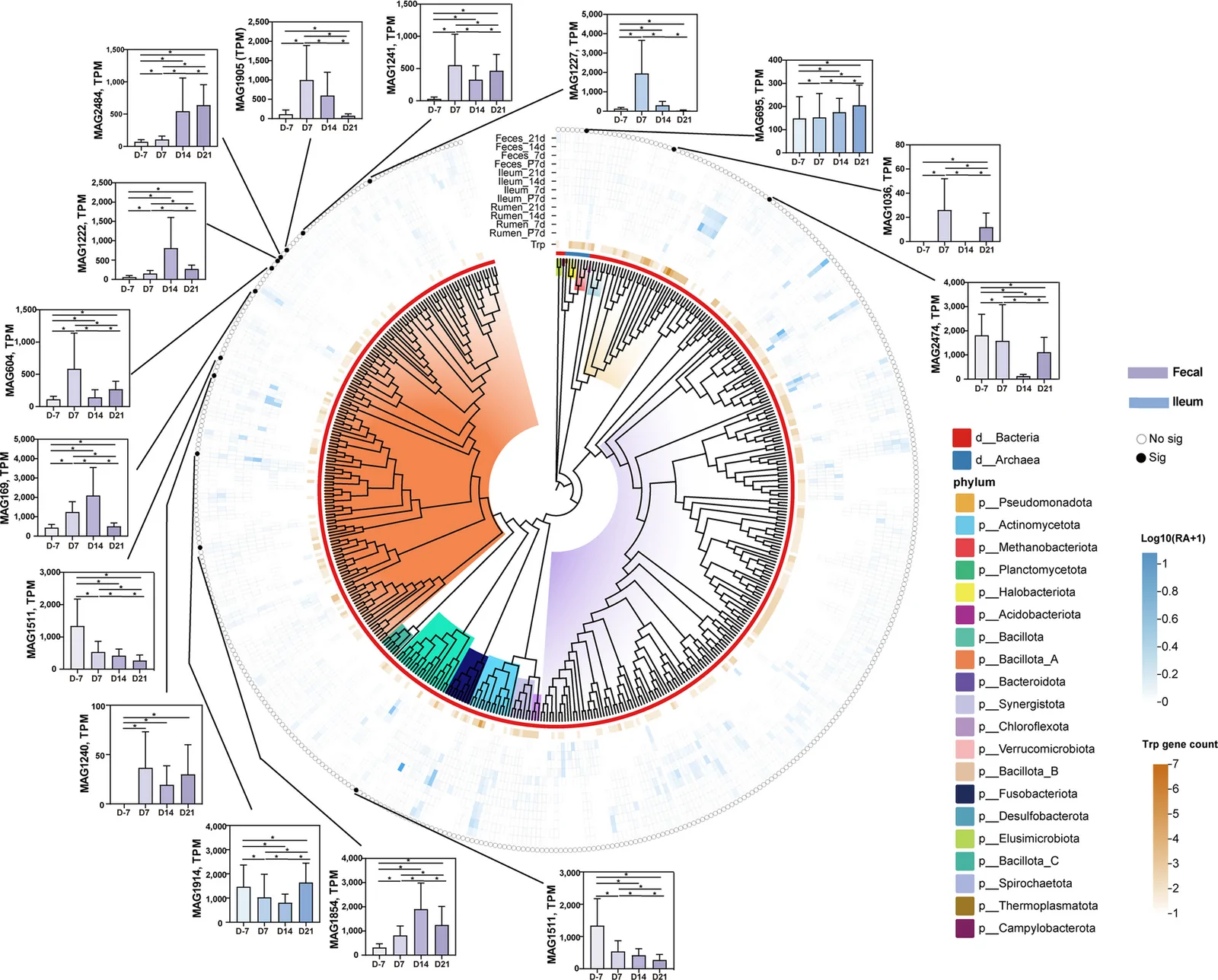
Phylogenetic tree of 461 metagenome-assembled genomes (MAGs) involved in tryptophan metabolism pathways sampled from the gastrointestinal of dairy cows. The clades are colored according to phylum. From inner to outer: the first strip charts show the domain-level affiliations of MAGs; the second strip charts display the number of tryptophan-metabolism genes present in each MAG; the heat map illustrates the distribution of MAGs across gastrointestinal regions and periparturient time points. Surrounding bar plots indicate the abundances of MAGs selected based on differential-abundance and correlation analyses. D−7, D7, D14, and D21 represent 7 d prepartum and 7, 14, and 21 d postpartum, respectively. ^*^P < 0.05

To gain insights into the microbes that mediate tryptophan metabolic pathways, we performed a taxonomic analysis of the genes involved in tryptophan metabolism. After quality evaluation using CheckM, 578 high-quality MAGs (completeness > 90% and contamination < 5%) were retained for subsequent analyses (Fig. S3). For annotation analysis, 461 MAGs encoding genes in the tryptophan metabolism pathways were identified (Fig. 6), including the kynurenine, 5-hydroxytryptamine, and indole pathways. The detailed annotation information and basic characteristics of these MAGs are provided in Table S5. The MAGs were assigned to 18 phyla, including Bacillota and Bacteroidete (Fig. S4), indicating that diverse microbes with tryptophan-metabolizing capacities are present in the intestines of dairy cows. To explore the association between MAGs encoding tryptophan metabolism genes and the host energy status, we analyzed their periparturient abundance dynamics and identified differentially abundant MAGs. Correlations between differential MAGs and tryptophan metabolites in each gastrointestinal region were evaluated (Table S6). Under the directionality criterion that metabolites were required to correlate positively with MAGs encoding upstream enzymes and negatively with MAGs encoding downstream enzymes, we identified two *acdA*-encoding and one *kynB*-encoding MAGs in the rumen, twelve *kynB*-encoding MAGs in feces, and none in the ileum (Fig. 6). We speculated that these MAGs are key drivers of periparturient gastrointestinal tryptophan metabolite production and fluctuations in host energy metabolism. We performed correlation analyses between the 13 genes involved in tryptophan metabolism and their metabolites. In the rumen, *acdA* was positively correlated with IPA, whereas no significant relationship was detected between *kynB* and its upstream or downstream metabolites (Fig. S5). In feces, *kynB* was significantly and inversely correlated with its upstream substrate, N-formylkynurenine. In addition, IPA concentration in the rumen, ileum, and plasma declined substantially during the postpartum period, and feces N-formylkynurenine levels decreased markedly. Next, we explored whether the decline in plasma IPA and KYN levels affected host energy metabolism. Therefore, we hypothesized that reduced dietary tryptophan intake or availability during the postpartum period lowers IPA in the rumen and ileum and decreases feces N-formylkynurenine, thereby reducing the systemic supply of IPA, KYN, and kynurenic acid. This might have contributed to the development of metabolic disturbances in dairy cows during the postpartum period.

### Modification of systemic metabolism by exogenous tryptophan supplementation in vivo

**Fig. 7:**
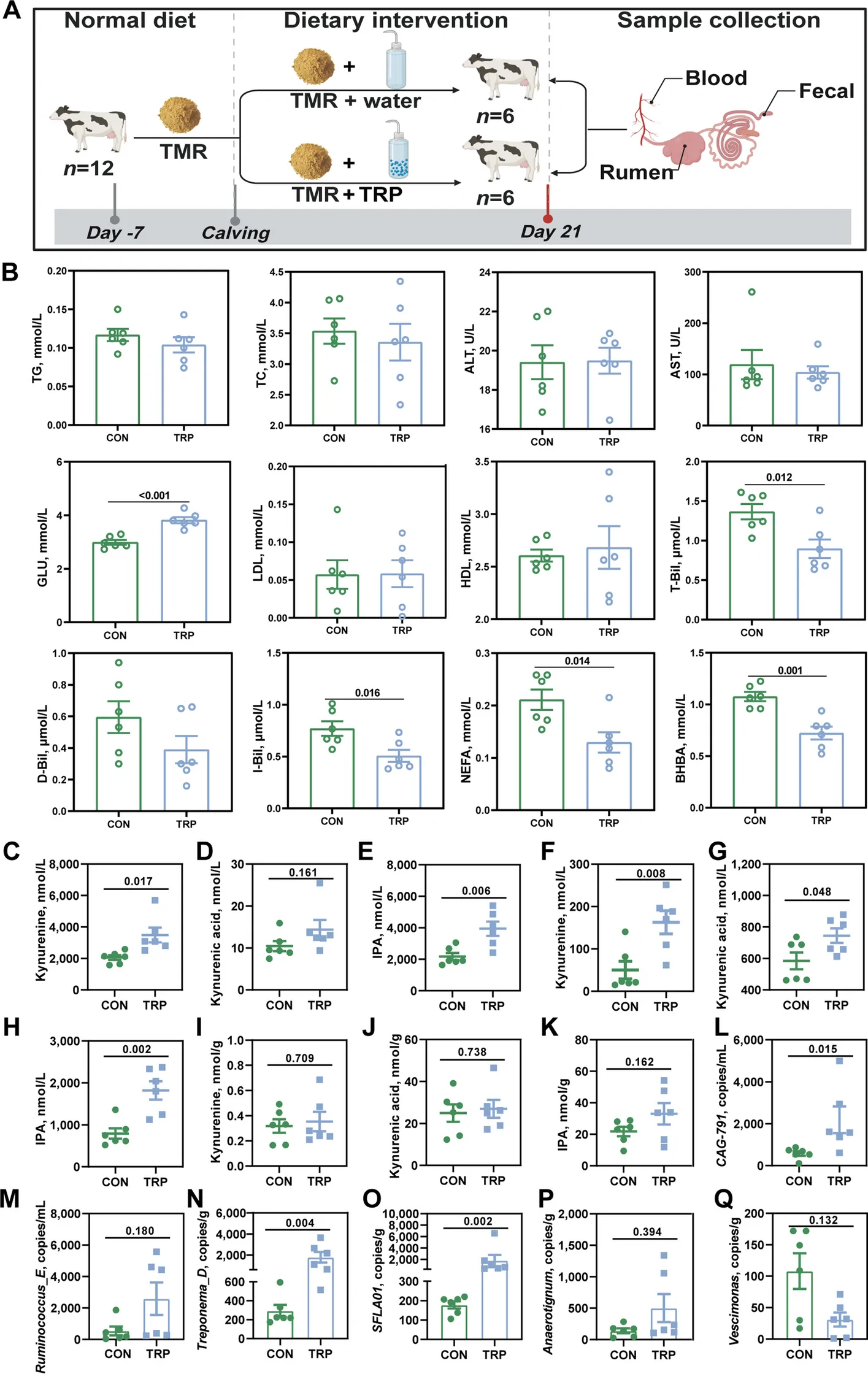
Exogenous tryptophan supplementation experiment. **A** Experimental design of the exogenous tryptophan supplementation experiment. **B** Effects of tryptophan supplementation on serum energy‐metabolism and liver function indices during the periparturient period, including β-hydroxybutyrate (BHBA), glucose (GLU), non-esterified fatty acids (NEFA), triglyceride (TG), total cholesterol (TC), aspartate aminotransferase (AST), alanine aminotransferase (ALT), low-density lipoprotein cholesterol (LDL), high-density lipoprotein cholesterol (HDL), blood urea nitrogen (BUN), total bilirubin (T-Bil), and direct bilirubin (D-Bil). Data are presented as mean ± standard error of the mean (SEM). **C–E** Concentrations of key tryptophan metabolites in plasma. **F–H** Concentrations of key tryptophan metabolites in the rumen. **I–K** Concentrations of key tryptophan metabolites in feces. **L **and** M** Absolute abundances of key bacterial genera in the rumen. **N–Q** Absolute abundances of key bacterial genera in feces

To further evaluate the effects of tryptophan supplementation in vivo, an in vivo supplementation experiment was conducted (Fig. 7A). No treatment effect was observed on TG, TC, ALT, AST, LDL, HDL, or D-Bil levels (*P* > 0.05). In contrast, relative to the controls, the tryptophan group showed lower NEFA, BHBA, T-Bil and D-Bil levels and higher GLU levels (*P* < 0.05; Fig. 7B). Collectively, these findings suggested that exogenous tryptophan lowers the risk of metabolic disturbances development in periparturient dairy cows. Additionally, as supplementary production-related indicators, milk yield and major milk components were evaluated on d 21 after calving. Milk yield, milk fat percentage, milk protein percentage, and lactose percentage did not differ significantly between the CON and TRP groups (*P* > 0.05; Table S7). To test whether concomitant increases in the metabolites IPA and KYN mediate this effect, we conducted targeted tryptophan metabolite profiling of rumen fluid, feces, and plasma.

**Fig. 8:**
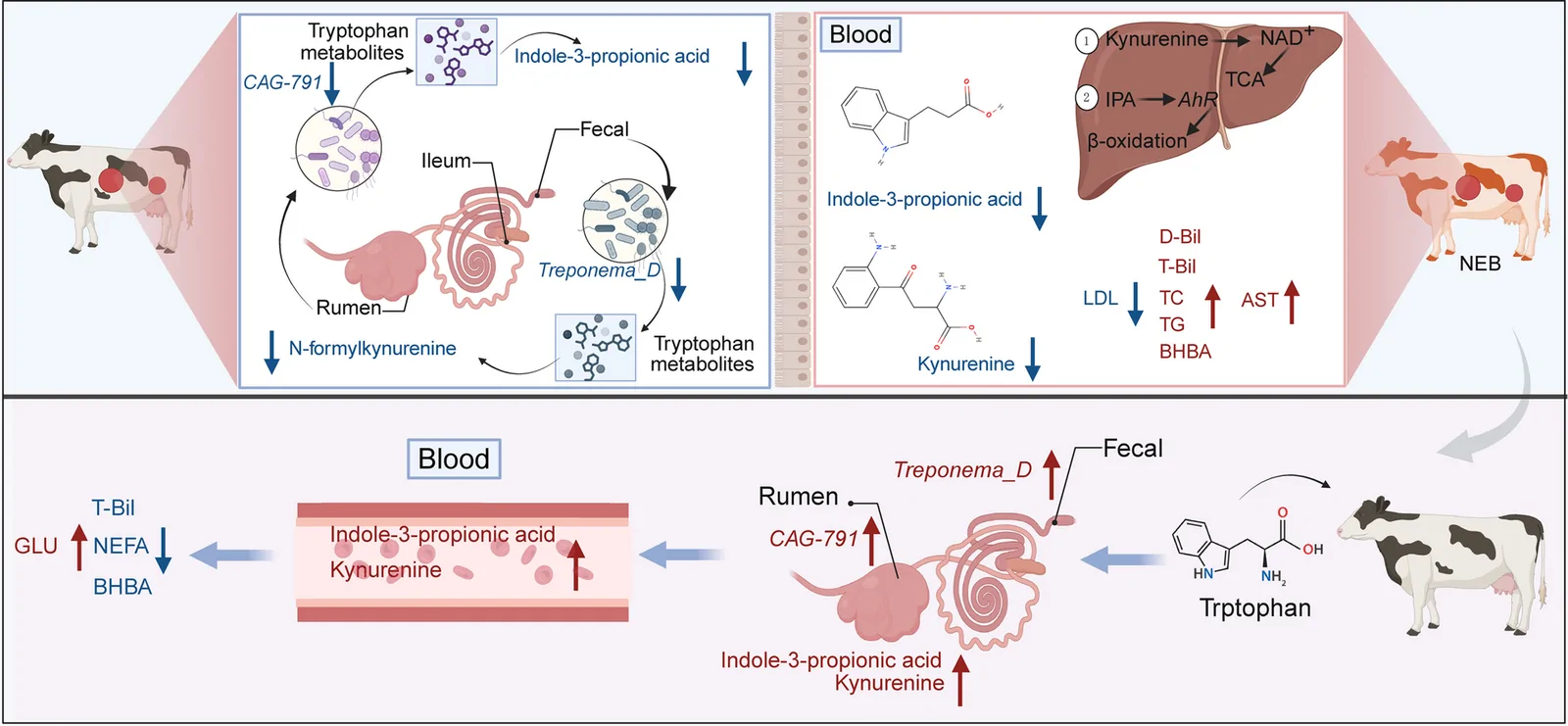
Conceptual model of tryptophan metabolic dysregulation in periparturient dairy cows and the improvement of metabolic adaptation by tryptophan supplementation. The upper panel summarizes the changes observed in periparturient dairy cows under postpartum metabolic challenge. Serum indicators related to energy metabolism and liver function were altered. In addition, gastrointestinal microbial tryptophan metabolism and host blood metabolic indicators changed markedly. Specifically, the tryptophan metabolism-associated rumen microbial taxon *CAG-791* and fecal microbial taxon *Treponema_D* were significantly decreased, together with reductions in several tryptophan metabolites in rumen fluid and feces. These changes were accompanied by decreased levels of indole-3-propionic acid and kynurenine in blood. The lower panel summarizes the potential effects of exogenous tryptophan supplementation. After tryptophan supplementation, the levels of the tryptophan metabolism-associated microbial taxa *CAG-791* in the rumen and *Treponema_D* in feces increased, accompanied by elevated blood levels of indole-3-propionic acid and kynurenine. Meanwhile, serum GLU increased, whereas metabolic burden-related indicators, including NEFA, BHBA, and T-Bil decreased. Blue downward arrows indicate decreases, and red upward arrows indicate increases. IPA, indole-3-propionic acid; AhR, aryl hydrocarbon receptor; NAD⁺, nicotinamide adenine dinucleotide; TCA, tricarboxylic acid cycle; GLU, glucose; NEFA, non-esterified fatty acids; BHBA, β-hydroxybutyrate; TG, triglyceride; TC, total cholesterol; LDL, low-density lipoprotein; AST, aspartate aminotransferase; T-Bil, total bilirubin; D-Bil, direct bilirubin

After tryptophan supplementation, the plasma concentrations of IPA and KYN increased significantly, whereas that of the downstream KYN metabolite kynurenic acid remained unchanged (Fig. 7C–E). Along the gastrointestinal tract, ruminal concentrations of IPA, KYN, and kynurenic acid were all elevated (Fig. 7F–H), whereas their levels in the feces did not change significantly (Fig. 7I–K). Taken together, these findings indicate that tryptophan supplementation mainly promotes tryptophan catabolism along the indole and kynurenine pathways, leading to key metabolite accumulation in the rumen and systemic circulation rather than their loss via feces excretion, thereby providing a metabolic basis for the alleviation of metabolic disturbances. To further identify the key microbial taxa mediating this metabolic remodeling, we performed full-length 16S rRNA gene amplicon sequencing combined with absolute quantification. This approach identified *CAG-791* and *Ruminococcus_E* as candidate genera in the rumen and *Treponema_D*, *SFLA_01*, *Anaerotignum*, and *Vescimonas* as candidate genera in feces (Fig. 7L–Q). Although several candidate genera showed region-specific responses, integration with our previous cannulated-cow experiment further narrowed the key taxa to *CAG-791* and *Treponema_D*. Specifically, *SFLA_01* was not significantly altered in the previous dataset, whereas *CAG-791* and *Treponema_D* were consistently different in the rumen and feces, respectively, and their changes closely mirrored the profiles of tryptophan-derived metabolites. Therefore, we designated *CAG-791* and *Treponema_D* as the primary candidate genera associated with ruminal and fecal tryptophan metabolism, respectively, and focused on them as the main targets in subsequent mechanistic studies (Fig. 8).

## Discussion

After calving, periparturient dairy cows often experience an energy deficit, and to meet the sharply increased energy demand during early lactation, they typically compensate by mobilizing body fat. This process is supported by concurrent changes in serum markers related to lipid mobilization and hepatic metabolic load. In the present study, serum BHBA levels markedly increased postpartum, indicating enhanced hepatic ketogenesis. Ketogenesis generally increases when the energy demand exceeds nutrient supply and fat mobilization accelerates during early lactation, reflecting heightened metabolic challenges during this period [47, 48]. This finding is consistent with previous studies reporting elevated serum BHBA levels in periparturient cows during early lactation [49]. Meanwhile, serum TG concentration was significantly higher on d 14 postpartum, suggesting an increased circulating lipid burden and greater hepatic demand for fatty acid uptake and processing [50]. Under sustained metabolic pressure, hepatocytes are prone to stress or injury, which is often accompanied by elevated AST levels. AST levels were consistently higher at d 7 postpartum than at d 7 prepartum and peaked at d 14 postpartum, exceeding the levels at d 7 and 21 postpartum. Previous work also indicates that elevated T-Bil (> 3.3 μmol/L) is indicative of impaired liver function, providing additional evidence for increased hepatic metabolic burden postpartum [51]. Collectively, the coordinated changes in BHBA, TG, AST, and T-Bil indicate that postpartum cows undergo marked metabolic fluctuations, accompanied by enhanced fat mobilization and increased hepatic metabolic stress.

Accordingly, we questioned whether various compounds produced by tryptophan metabolism can help alleviate the metabolic fluctuations. We systematically profiled tryptophan-related metabolites across the gastrointestinal segments and plasma of periparturient cows and observed pronounced site-by-time fluctuations, revealing marked periparturient shifts in key metabolites, notably IPA and KYN. Specifically, during the postpartum period, the IPA concentration declined significantly in the rumen, ileum, and plasma, whereas KYN and its downstream product kynurenic acid decreased significantly in the rumen and plasma. This aligns with prior findings indicating that tryptophan metabolism is a significantly altered pathway in cows with ketosis compared to healthy individuals. Specifically, cows with ketosis exhibit significant reductions in the levels of tryptophan, KYN, and indole derivatives compared with healthy controls [19, 52]. Cows with high ketonemia often exhibit tryptophan deficiency [53, 54]. Therefore, KYN has been proposed as a novel postpartum biomarker of metabolic disturbances [55]. We conducted correlation analyses between the microbiota from individual gastrointestinal segments and serum biochemical traits. Given that BHBA and TG are established indicators of energy status, we used them as reference indices and observed significant negative correlations between plasma KYN and BHBA, suggesting kynurenine pathway suppression under severe metabolic disturbances. Notably, this result mirrors previous research on transitionally ketotic cows, confirming the inverse relationship between KYN and BHBA [56]. Furthermore, the observed inverse correlation between plasma IPA and TG levels highlights the potential role of gut-derived tryptophan metabolites in the modulation of lipid mobilization. Although this specific mechanism has not yet been directly verified in perinatal cows, extensive studies in mice provide supporting evidence. IPA activates the aryl hydrocarbon receptor (*AhR*), which downregulates key hepatic lipogenic genes (e.g., *SREBP-1c*), thereby significantly reducing TG levels in both the liver and plasma [57]. Collectively, our findings support the notion that tryptophan metabolism is disrupted during the postpartum period, with synchronized decreases in tryptophan and its metabolites in the plasma and gastrointestinal. Multiple tryptophan metabolites are closely associated with metabolic disturbances.

Tryptophan metabolism in vivo depends on gut microbiota [58]. Given the pronounced microbial heterogeneity and metabolic partitioning across gastrointestinal segments in ruminants [59–61], we performed segment-resolved metagenomic profiling in periparturient dairy cows and subsequently performed assembly and binning to reconstruct high-quality MAGs. Notably, metagenomic analysis demonstrated a distinct metabolic preference of the gut microbiota for tryptophan utilization. Most annotated tryptophan metabolism genes belong to the indole and kynurenine pathways, whereas microbial genes involved in serotonin synthesis are scarce. This finding is consistent with previous knowledge: within the context of host-microbe interactions, tryptophan is often converted by the microbiota into indole-derived metabolites or funneled into kynurenine-related metabolic branches, and both classes of products have been repeatedly linked to host energy metabolism and inflammatory homeostasis [62–66]. Together with the distribution of functional genes observed across different gastrointestinal segments in our study, these results suggest that, during the periparturient period, microbial tryptophan utilization may be preferentially directed toward these two branches, thereby contributing to the metabolic remodeling.

After metagenomic assembly and binning, we reconstructed 578 high-quality MAGs, of which 461 contained genes involved in tryptophan metabolism. Gene-bearing MAGs were mainly assigned to Bacillota_A, Pseudomonadota, and Bacteroidota, consistent with reports that the dominant gut producers of indole and indole derivatives and key enzymes of the kynurenine pathway were enriched in lineages affiliated with Firmicutes, Proteobacteria, and Bacteroidota [67–69]. To focus on microbial regulation, we screened functional groups that varied over time and were significantly associated with tryptophan-derived metabolites in a segment-resolved longitudinal dataset, finally, we identified two ruminal MAGs harboring *acdA*, which drives IPA formation, and one ruminal MAG harboring *kynB*, as well as twelve feces MAGs harboring *kynB*, which catalyzes the N-formylkynurenine—KYN step. These findings pinpoint *acdA*- and *kynB*-centered functional modules at the levels of taxa, genes, and metabolites.

To test this hypothesis, we conducted a postpartum tryptophan supplementation trial and measured serum biochemical indicators closely related to metabolic status. Compared with the control group, cows receiving tryptophan supplementation had higher serum glucose concentrations, which is consistent with previous findings showing that pulse dosing with tryptophan increases circulating glucose [70]. These results suggest that tryptophan supplementation may help improve the postpartum energy metabolic status. Another study reported that tryptophan supplementation reduced NEFA and BHBA levels at postpartum time points and was accompanied by improved antioxidant and inflammatory indices, indicating reduced lipid mobilization and ketogenesis [71]. A similar pattern was observed in our study, as tryptophan supplementation significantly decreased NEFA, BHBA, and T-Bil levels. These findings are consistent with those of previous reports and indicate that exogenous tryptophan administration during the periparturient period can alleviate metabolic disturbances and reduce hepatobiliary and lipolytic load [72]. Collectively, our intervention trial provides nutritional evidence supporting the conclusion that insufficient tryptophan availability during the periparturient period may aggravate metabolic disturbances during the postpartum period, whereas dietary tryptophan supplementation may help alleviate this condition.

At the level of tryptophan metabolites, the pattern of increased IPA and KYN in the rumen and plasma, with no change in feces, suggests that tryptophan supplementation mainly promotes IPA and KYN accumulation in the rumen and systemic circulation, rather than their loss via feces excretion. This spatial distribution was consistent with the mode of action by which microbial tryptophan metabolites regulate systemic metabolism. The above results are consistent with previous inferences, indicating that supplementing cows with tryptophan during the late perinatal period increases microbial-derived IPA and KYN metabolic flux. As a gut microbiota-derived tryptophan metabolite, IPA can be absorbed and delivered to the liver via the portal circulation [73]. In the liver, IPA activates the *AhR* and downregulates lipogenic genes, thereby suppressing hepatic lipid accumulation and metabolic dysregulation [74–76]. In ruminants, evidence indicates that the ruminal epithelium absorbs indole derivatives into the bloodstream [77, 78]. Moreover, the AhR pathway is present in the liver and can be activated [79, 80], providing a physiological basis for IPA action. However, whether IPA modulates gluconeogenesis in cow hepatocytes via *AhR* requires further validation using in vitro hepatocyte culture. Meanwhile, the KYN pathway is the key route for de novo NAD⁺ synthesis from tryptophan, with the liver serving as the primary site of NAD⁺ production [66]. Given that NAD(H) functions as a central electron carrier in the TCA cycle and mitochondrial oxidative metabolism, changes in NAD availability may influence hepatic oxidative flux and ketone body metabolism [81, 82]. In addition, studies on dairy cows have shown that niacin supplementation (an NAD precursor) reduces circulating BHBA and NEFA [83–85], supporting an association between NAD-related metabolic status and the ketotic phenotype. These mechanisms require direct validation in ruminants by measuring the hepatic NAD pools and metabolic fluxes. Therefore, MAGs harboring *acdA* and *kynB*, together with IPA and KYN, constitute priority targets for intervention.

At the microbiota level, we further investigated the taxa that might drive metabolic remodeling. Based on full-length 16S rRNA sequencing with absolute quantification, combined with our previous longitudinal data, only *CAG-791* in the rumen and *Treponema_D* in the feces showed significant increases in abundance after tryptophan supplementation, and their temporal patterns closely matched the dynamics of key metabolites, such as IPA and KYN. This covariation of phenotypes, metabolites, and microbial taxa highlighted these two groups among the many candidates. *CAG-791* belongs to the class Clostridia, which includes several experimentally confirmed IPA-producing bacteria [58, 68]. For example, *Clostridium sporogenes* carries a complete tryptophan-indole-IPA conversion pathway and can markedly alter IPA levels in host tissues [86, 87]. In this context, our observation that *CAG-791* abundance and IPA levels increased after tryptophan supplementation suggests that *CAG-791* may be an essential node in the rumen IPA-producing network. *Treponema_D* was assigned to the genus *Treponema* within the phylum Spirochaetota. Several representative Treponema strains have been identified as carrying tryptophan metabolism genes and can convert tryptophan into indole and related derivatives [88, 89]. In the present study, *Treponema_D* in fecal samples correlated with KYN, and both *Treponema_D* and KYN increased after tryptophan supplementation. These findings support that *Treponema_D* may participate in the regulation of the hindgut tryptophan-kynurenine pathway, help maintain NAD⁺ supply, ultimately contributing to the alleviation of metabolic disturbances [90]. These taxa provide clear targets for future isolation, culture-based studies, and causal validation.

During the periparturient period, increasing tryptophan supplementation in the rumen selectively enriches indole-producing microbes such as *CAG-791* and enhances the hindgut kynurenine pathway flux represented by *Treponema_D*. This remodeling alleviated metabolic disturbances. Our results indicate that tryptophan insufficiency aggravates postpartum metabolic disturbances and that dietary tryptophan supplementation alleviates this condition. This study had several limitations. First, although *CAG-791* and *Treponema_D* showed concordant changes in absolute abundance and phylogenetic evidence, we did not isolate representative strains or directly validate their metabolic capacity. Second, although IPA and KYN have been implicated in the alleviation of metabolic disturbances, the mechanistic pathways linking these metabolites to hepatic energy metabolism require further validation. Additionally, it is important to acknowledge that due to the technical challenges and intensive postoperative care required for cannulated cows, the sample size in this study was necessarily limited. Future studies should strengthen the causal inferences through additional in vivo and in vitro experiments, including strain isolation and culture-based validation, functional characterization of key genes, and targeted intervention trials with relevant metabolites.

## Conclusions

In this study, periparturient dairy cows fitted with rumen and ileal cannulas were used as an in vivo model. The results showed that metabolic disturbances were not only characterized by classical abnormalities in BHBA and TG, but also by a state marked by broadly reduced levels of IPA and KYN in the rumen, ileum, and plasma. Moreover, our results provide strong evidence that the ruminal, ileal, and feces microbiota of periparturient cows can convert tryptophan into multiple metabolites. We systematically profiled gastrointestinal tryptophan metabolites and identified 461 high-quality MAGs carrying genes involved in tryptophan metabolic pathways, thereby establishing a key role of the cow gut microbiome in this process. Metagenomic analyses further showed that ruminal Clostridia taxon *CAG-791* harbors *acdA* and contributes to IPA production. In contrast, the hindgut taxon *Treponema_D* harbors *kynB* and promotes KYN formation, which defines two critical microbe-metabolite regulatory nodes. In a tryptophan supplementation trial, increased blood glucose and decreased T-Bil, NEFA, and BHBA, together with targeted increases in ruminal and plasma IPA and KYN, functionally validated the “tryptophan-*CAG-791*/*Treponema_D*-IPA/KYN” axis in alleviating metabolic disturbances during the periparturient period. These findings provide a mechanistic basis for developing strategies targeting tryptophan metabolism and key microbial taxa to improve energy metabolism and liver health in dairy cows.

## Supplementary Information

Additional file 1: Fig. S1. Random forest analysis of targeted tryptophan metabolites across intestinal segments and plasma. Fig. S2. Distribution of genes involved in tryptophan metabolism. Fig. S3. Phylogenetic tree of 578 high-quality metagenome-assembled genomes (MAGs) from the intestine of dairy cows. Fig. S4. Phylogenetic distribution of tryptophan metabolism genes at the phylum level across three intestinal segments and four periparturient time points. Fig. S5. The associations between the genes encoding tryptophan metabolism pathway and tryptophan metabolites.

Additional file 2: Table S1. The feed ingredients and nutrient composition of diets in the cannulated cow study. Table S2. The feed ingredients and nutrient composition of diets in tryptophan supplementation experiment. Table S3. Summary of sequencing statistics for metagenomic samples. Table S4. Spearman correlations between significantly altered serum biochemical indices and tryptophan metabolites across gastrointestinal sites. Table S5. Taxonomic classification and genomic quality metrics of 461 MAGs harboring tryptophan metabolism-related genes. Table S6. Spearman correlations between differential MAGs and significantly altered tryptophan metabolites across gastrointestinal sites. Table S7. Milk yield and milk component composition of cows in the CON and TRP groups on d 21 after calving.

## Data Availability

The metagenomic data from rumen, ileum and feces in this study were uploaded to the NCBI Sequence Read Archive (SRA) under the accession number PRJNA1363106, PRJNA1359591, PRJNA1359117, respectively. The full-length 16S rRNA gene amplicon sequencing data from the tryptophan supplementation experiment were uploaded to the NCBI Sequence Read Archive (SRA) under the accession number PRJNA1372445. The metabolomics data have been deposited in NGDC OMIX repository (PRJCA057629).

## Abbreviations

ALT: Alanine aminotransferase
AST: Aspartate aminotransferase
ASV: Amplicon sequence variant
BHBA: β-Hydroxybutyrate
BUN: Blood urea nitrogen
D-Bil: Direct bilirubin
GLU: Glucose
HDL: High-density lipoprotein
IPA: Indole-3-propionic acid
KYN: Kynurenine
LDL: Low-density lipoprotein
MAGs: Metagenome-assembled genomes
NEFA: Non-esterified fatty acids
TC: Total cholesterol
T-Bil: Total bilirubin
TG: Triglyceride
