# Supplementary material for: Gut microbiota-driven indole-3-propionic acid and kynurenine production is associated with improved metabolic adaptation in periparturient dairy cows
Source: J Anim Sci Biotechnol. 2026 Sep 11;17:179. doi: 10.1186/s40104-026-01497-6 (PMC13563733; doi:10.1186/s40104-026-01497-6)
Supplement: Supplementary file 1 [file 40104_2026_1497_MOESM1_ESM.pdf]

Additional file 1:

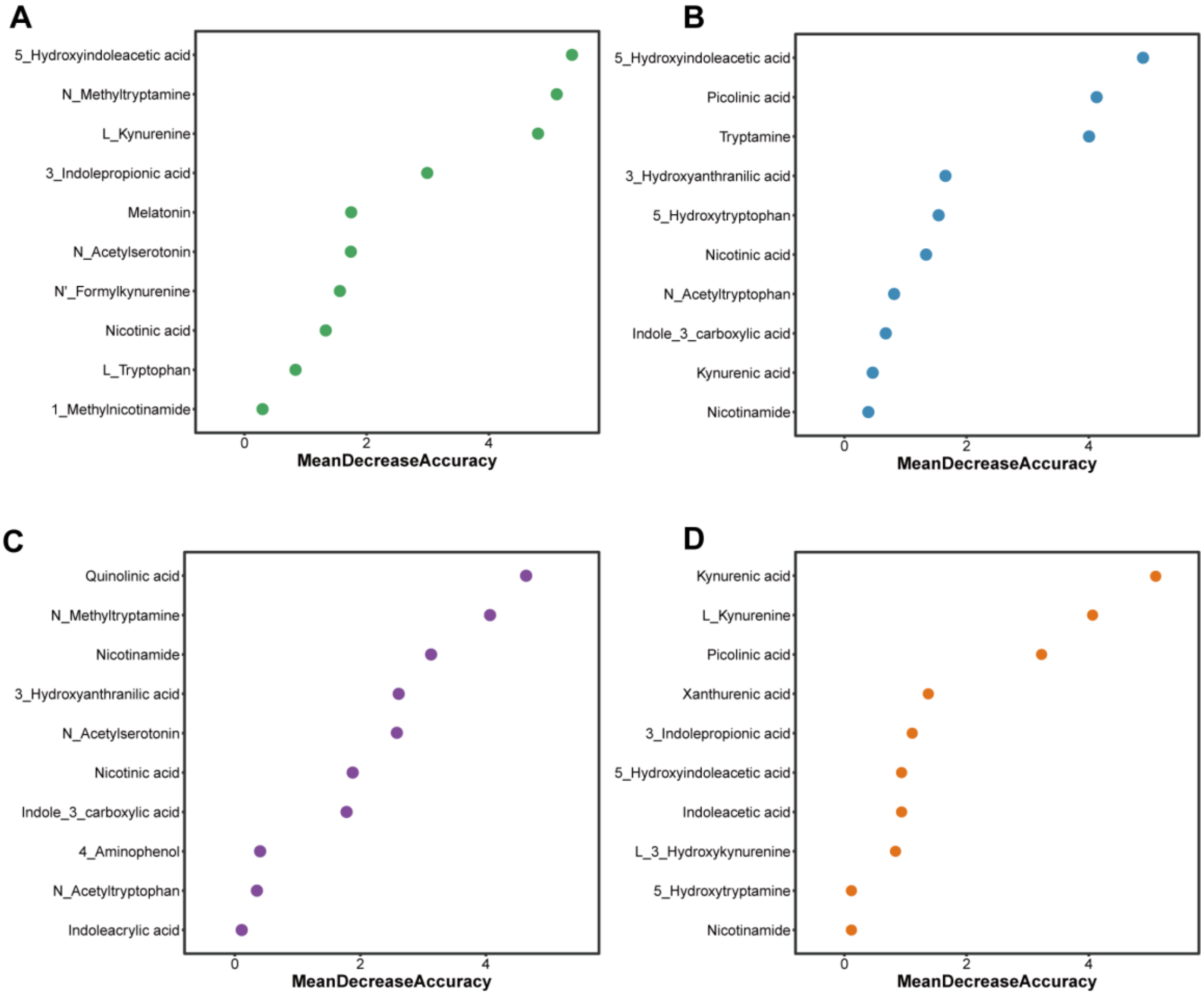

Fig. S1 Random forest analysis of targeted tryptophan metabolites across intestinal segments and plasma. **A** rumen; **B** ileum; **C** feces; **D** plasma. The x-axis shows MeanDecreaseAccuracy, which represents the decrease in classification accuracy when a given metabolite is permuted and therefore indicates its relative contribution to the model. Each circle represents one metabolite, and circles positioned farther to the right indicate higher variable importance in the random forest model.

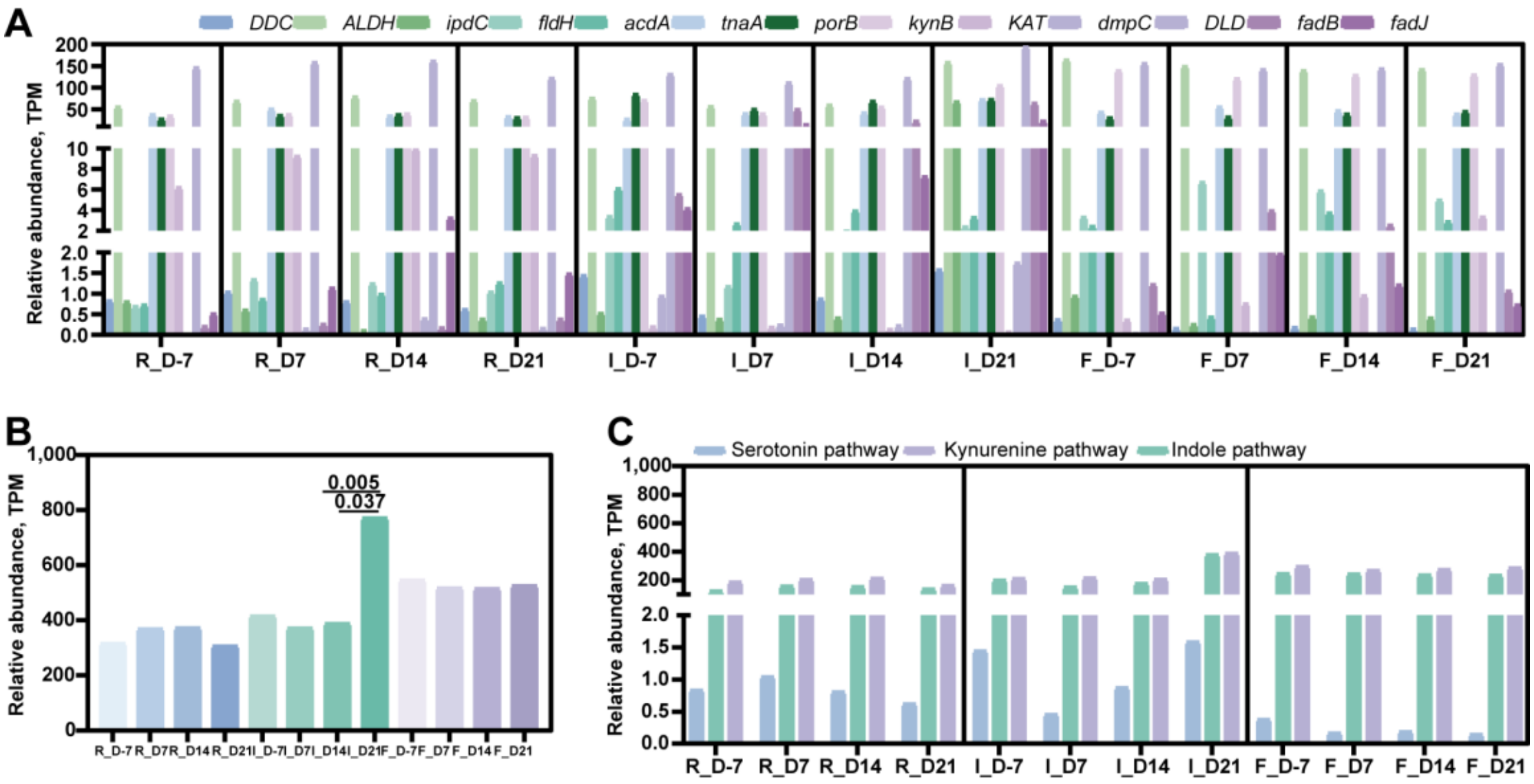

Fig. S2 Distribution of genes involved in tryptophan metabolism. **A** Distribution of the thirteen pathway genes across intestinal segments and sampling time points. **B** Overall distribution of pathway genes across intestinal segments and sampling time points. **C** Overall distribution of genes in the three tryptophan metabolism pathways across segments and time points. R, I, F, and P represent rumen fluid, ileal digesta, feces, and plasma, respectively, whereas D-7, D7, D14, and D21 represent 7 d before calving and 7, 14, and 21 d after calving, respectively.

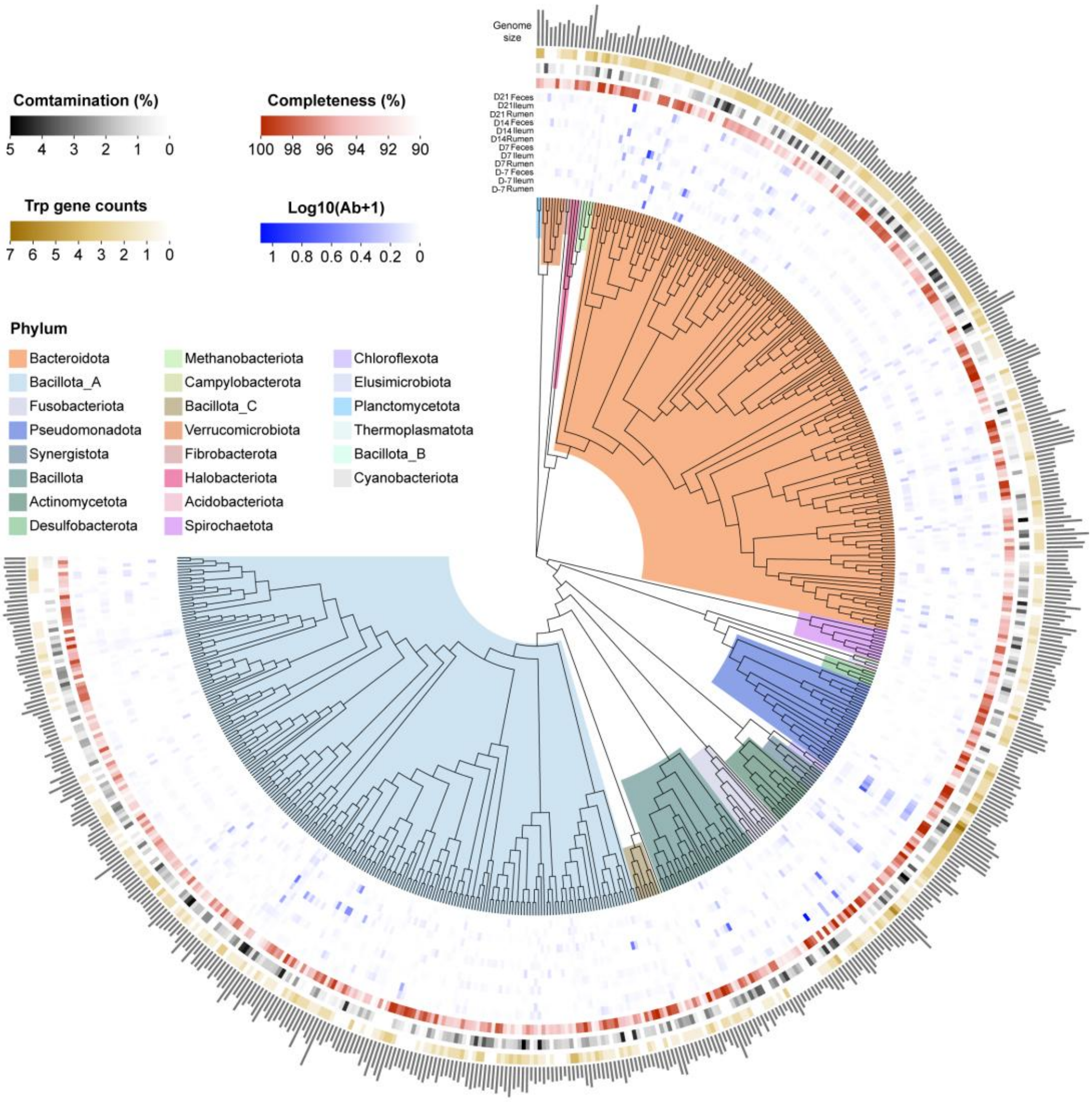

Fig. S3 Phylogenetic tree of 578 high-quality metagenome-assembled genomes (MAGs) from the intestine of dairy cows. The clades are colored according to phylum. The heatmap illustrates the distribution of MAGs across intestinal regions and periparturient time points. Concentric annotation tracks, from inner to outer: genome completeness; genome contamination; tryptophan-metabolism gene count per MAG and genome size. D-7, D7, D14, and D21 represent 7 d prepartum and 7, 14, and 21 d postpartum, respectively.

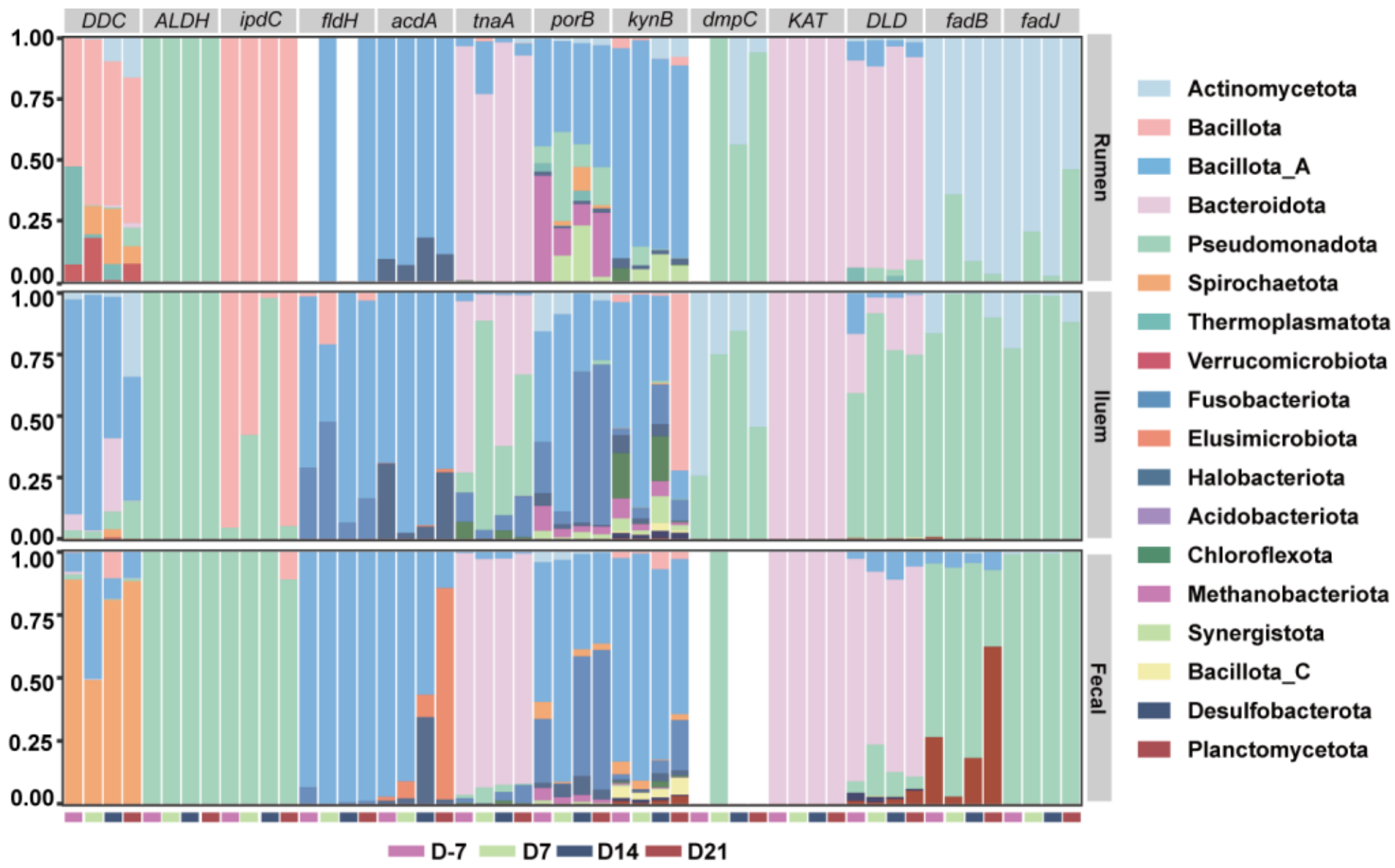

Fig. S4 Phylogenetic distribution of tryptophan metabolism genes at the phylum level across three intestinal segments and four periparturient time points. D-7, D7, D14, and D21 represent 7 d prepartum and 7, 14, and 21 d postpartum, respectively.

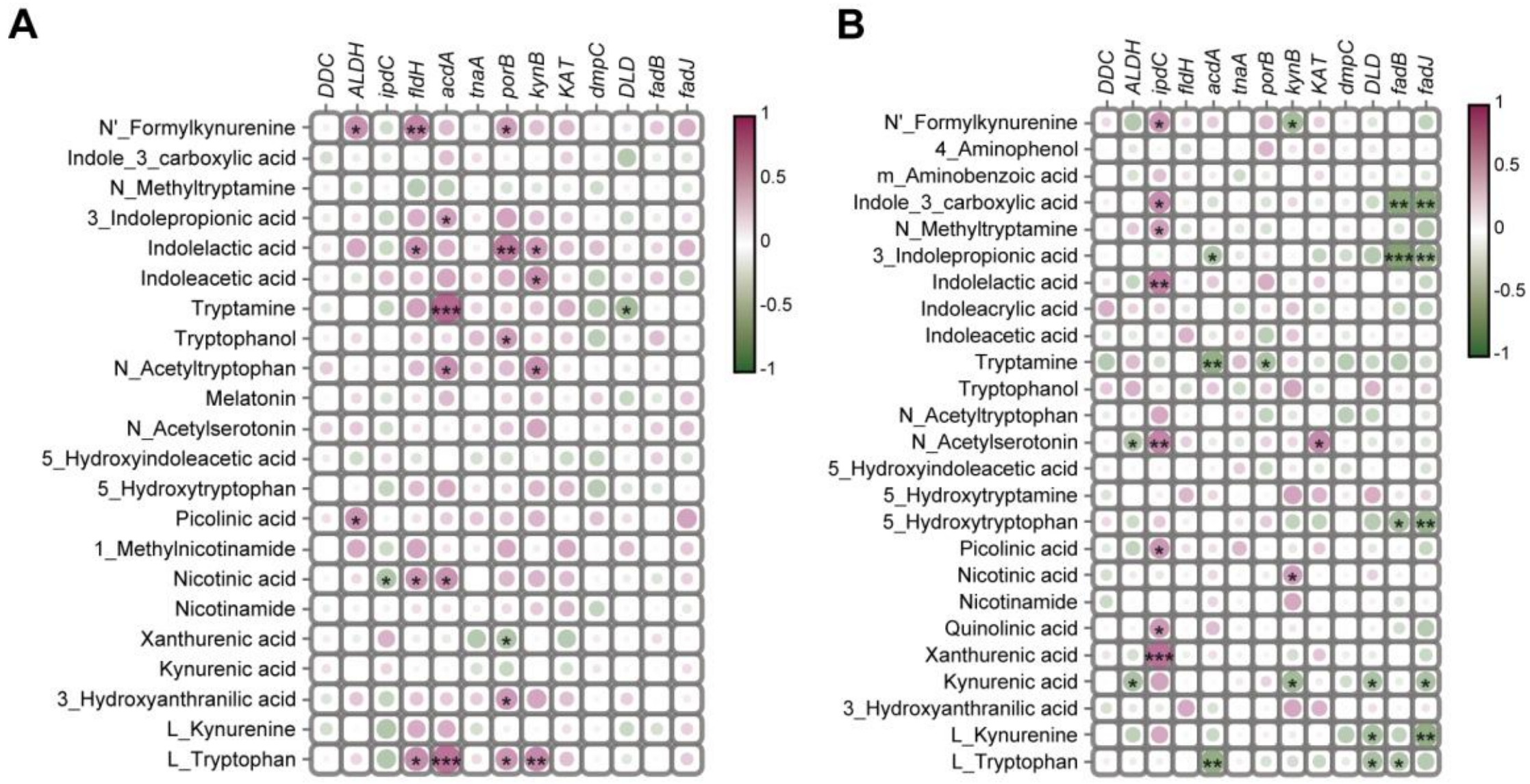

Fig. S5 The associations between the genes encoding tryptophan metabolism pathway and tryptophan metabolites. **A** Correlations between the abundances of genes encoding tryptophan metabolism in ruminal microbiota and ruminal tryptophan metabolites. **B** Correlations between the abundances of genes encoding tryptophan metabolism in feces microbiota and feces tryptophan metabolites. *$P < 0.05$; **$P < 0.01$; ***$P < 0.001$.
